# Prevalence of depression in patients with sarcopenia and correlation between the two diseases: systematic review and meta‐analysis

**DOI:** 10.1002/jcsm.12908

**Published:** 2022-01-08

**Authors:** Zhenzhen Li, Xiang Tong, Yao Ma, Ting Bao, Jirong Yue

**Affiliations:** ^1^ Health Management Center, National Clinical Research Center for Geriatrics, West China Hospital/West China School of Medicine Sichuan University Chengdu China; ^2^ Department of Respiratory and Critical Care Medicine, West China Hospital/West China School of Medicine Sichuan University Chengdu China; ^3^ Department of Geriatrics and National Clinical Research Center for Geriatrics, West China Hospital/West China School of Medicine Sichuan University Chengdu Sichuan China

**Keywords:** Sarcopenia, Depression, Prevalence, OR

## Abstract

**Background:**

Depression may be the most common cause of emotional distress later in life and can significantly reduce the quality of life in elderly individuals. Sarcopenia is a syndrome characterized by the continuous loss of skeletal muscle mass and decreased strength and function. In recent years, many studies have shown a correlation between sarcopenia and depression. The present study aimed to investigate the prevalence of depression among individuals with sarcopenia and to ascertain whether sarcopenia is independently associated with depression.

**Methods:**

We systematically searched the PubMed, Embase, and Google Scholar databases for papers on sarcopenia published up to 31 August 2021. We reviewed the literature on the number of individuals with sarcopenia, the number of individuals with both sarcopenia and depression, and the odds ratio (OR) of sarcopenia to depression. Statistical analyses were performed using Meta‐DiSc 1.4 software and Stata version 12.0.

**Results:**

Nineteen articles met the inclusion criteria for review: nine reported both prevalence and ORs, four described prevalence only, and six detailed the ORs only. Regarding prevalence, there were 1476 cases of sarcopenia and 364 of depression in the selected studies; the mean age of the patients was 75.5 years, and the overall prevalence of depression was 0.28 [95% confidence interval (CI): 0.21–0.36]. Significant heterogeneity was noted (*P* < 0.001; *I* ^2^ = 92.2%). In the case of ORs, there were 16 869 subjects with a mean age of 73 years; the overall adjusted OR between sarcopenia and depression was 1.57 (95% CI: 1.32–1.86). Significant heterogeneity was noted in the adjusted ORs (*P* < 0.001; *I* ^*2*^ = 75.1%).

**Conclusions:**

The prevalence of depression in patients with sarcopenia was high relatively, and there was a correlation between sarcopenia and depression.

## Introduction

Depression may be the most common cause of emotional distress later in life; it affects work, significantly reduces the quality of life, increases mortality and cardiovascular disease risk, and is related to poor health and suicide in older people.^1^, ^2^ Suicide due to depression is a major public health problem; every year, more than 800 000 people worldwide die by suicide.^3^ The prevalence of clinically relevant depressive symptoms in the elderly population in Asia ranges from 8% to 13%.^4^ Major depressive disorder (MDD) is a common chronic disease that affects more than 300 million people; it is the main cause of disability worldwide and the main factor contributing to the overall global burden of disease.^5^ In the United States, approximately 10.4% of adults have had MDD in the past year, and 20.6% of adults have experienced MDD in their lifetime.^6^ In particular, depression in the elderly has become a major problem, with high prevalence and significant relationships to other adverse health events.^7^ In countries of all income levels, people with depression are often diagnosed late or incorrectly; therefore, it is important to screen early and intervene before the disease becomes serious. Many studies have found that depression is related to body composition,^8^, ^9^, ^10^, ^11^ as well as to skeletal muscle mass, strength, and function, which are important to vitality and well‐being; relatedly, patients with sarcopenia are more likely to suffer from depression and have a higher mortality rate.^12^

Sarcopenia, defined as age‐related loss of muscle mass, muscle strength, and/or physical function,^13^ can easily lead to fractures and joint damage, affect organ function, and progress to cardiopulmonary failure and even death.^14^ It is an important contributor to dysfunction and disability, affecting quality of life and increasing morbidity and mortality.^13^, ^14^, ^15^, ^16^, ^17^ The prevalence of sarcopenia is 5–13% in individuals aged 60–70 years, while it varies from 11% to 50% in those over 80 years old.^18^ A conservative estimate of the prevalence of clinically relevant sarcopenia is that the syndrome currently affects more than 50 million individuals, and this number is expected to surpass 200 million in the next 40 years.^13^ Sarcopenia is associated with several major economic and social impacts, and the direct cost of sarcopenia has accounted for 1.5% of total healthcare expenditure in recent years.^19^ Effective treatments for sarcopenia include physical exercise, nutritional intervention, and hormone therapy. Early and timely treatment can alleviate the condition and reduce complications.

As two common diseases in the elderly, sarcopenia and depression have some similarities in clinical, aetiology, and prognosis. In recent years, a number of studies have shown a correlation between sarcopenia and depression. There are several common risk factors for both, such as lack of exercise, upregulation of inflammatory factors, and hormonal disorders of the hypothalamic–pituitary–adrenal axis.^20^ By calculating the prevalence and correlation of depression in sarcopenia, we can calculate the sample size in scientific research and provide a basis for future prospective research. In clinical practice, this knowledge can increase the vigilance of clinicians on depression in patients with sarcopenia so that the disease can be diagnosed and treated at its earliest stages, prevent disease progression and complications, improve the quality of life of patients, and reduce the economic burden on society.

However, the latest studies, based on observational research data, have demonstrated wide‐ranging and inconsistent results. Studies of depressive symptoms in older people with sarcopenia have reported a prevalence of 8% to 87%. Furthermore, the association between sarcopenia and depression is inconsistent. Some studies have reported that sarcopenia is positively associated with depression [odds ratio (OR) 6.87, 95% confidence interval (CI): 2.06–22.96].^21^ Others have reported an OR of 0.82 (95% CI: 0.5–1.36),^22^ showing no statistically significant association between sarcopenia and depression. There is no systematic review of the prevalence of depression in sarcopenia and only one meta‐analysis on the correlation between them, but that meta‐analysis confused pre‐sarcopenia and sarcopenia because the diagnosis of sarcopenia was inconsistent in the included studies. All studies included in the present meta‐analysis were published before 2016.^23^ Thus, most research on the correlation between sarcopenia and depression has been conducted in the past 3 years. This meta‐analysis aimed to explore the prevalence of depression in patients with sarcopenia and to ascertain whether sarcopenia is independently associated with depression.

## Methods

### Study selection

We followed the principles of the Preferred Reporting Items for Systematic Reviews and Meta‐Analyses 2020 statement (PRISMA 2020). Two reviewers searched PubMed, Embase, and Google Scholar for meta‐analyses related to the prevalence of depression in sarcopenia, but no such articles were found. A more comprehensive search was conducted in the same three electronic databases to identify eligible studies published up to August 31, 2021. The following search terms were used: ‘sarcopenia’ OR ‘muscle loss’ OR ‘grip strength’ OR ‘gait speed’ AND ‘depression’ OR ‘depressive symptom.’ Retrieve the formula: ((((sarcopenia) OR (muscle loss)) OR (grip strength)) OR (gait speed)) AND (depression)) OR (depressive symptom). We also screened the reference lists of all retrieved articles to identify other relevant research.

### Inclusion and exclusion criteria

All included studies met the following criteria: (1) cross‐sectional, cohort study design; (2) study population involving individuals with sarcopenia, defined as the presence of low muscle mass (LMM), low muscle strength (LMS), and/or low physical performance (LPP). All studies calculating prevalence included patients with sarcopenia, while all those calculating the correlation included a case group of patients with both sarcopenia and depression and a control group of patients with sarcopenia, but no depression (3) clear diagnostic criteria for depression; (3) outcomes of prevalence of depression and ORs. The exclusion criteria were as follows: (1) inability to extract data; (2) articles not written in English; and (3) case reports, letters to the editor, abstracts, and review articles.

### Data extraction

Two reviewers independently assessed the studies' eligibility according to the inclusion/exclusion criteria. In cases of disagreement, they consulted until a resolution was reached. A standard procedure was performed to extract the data from the studies, including the first author, country/region, publication year, study type, sample size, age, number of patients with sarcopenia, number of patients with depression among those with sarcopenia, methods of evaluating sarcopenia and depression, including diagnostic items, techniques for measurement, and cut‐off values, and all pertinent covariates modifying the relationship between sarcopenia and depression. The main results of interest were the prevalence of depression in sarcopenia and the rough and adjusted association between sarcopenia and depression, expressed in odds ratios (ORs) and 95% CIs. Results were adjusted for different confounding factors; if multiple logistic regression models were produced, the most common one was chosen.

### Research quality assessment

The quality of each study was independently scored by the two researchers and assessed using the Newcastle–Ottawa Scale, which is widely used to evaluate cross‐sectional studies. The highest score for cohort or case–control studies was 9 points, and the highest score for cross‐sectional studies was 6 points.^24^ A higher score indicates a better quality method. Disagreements between the two researchers were resolved through discussion.

### Statistical analyses

We followed the statistical analysis methods of Tong *et al*.^25^ All data analyses were performed using STATA 12.0 (StataCorp LLC, Texas, USA, http://www.stata.com/). After proper conversion, we chose random effects analysis because it is considered more conservative, combines better interstudy variance terms, produces a lower Type I error rate, has a wider CI than the fixed‐effects model, and summarizes the effect estimates.^26^ Heterogeneity between studies was investigated using the Q test, based on chi‐square (*χ* ^2^) and the statistical test of *I* ^2^. A *P* value of less than 0.10 or an *I* ^2^ value greater than 50% indicated statistical heterogeneity.

### Subgroup analysis

To investigate the possible reasons for heterogeneity, we performed subgroup analyses on the diagnostic criteria for sarcopenia, the diagnostic items of sarcopenia and depression, body mass index (BMI), ethnicity, and study site. A BMI of ≥25 was defined as overweight, while one ≥30 was considered obese.^27^

### Sensitivity analysis

If a sufficient number of studies were found, we intended to evaluate the robustness of the results by sensitivity analysis, excluding studies conducted in special populations.

## Results

### Search process

**Figure 1:**
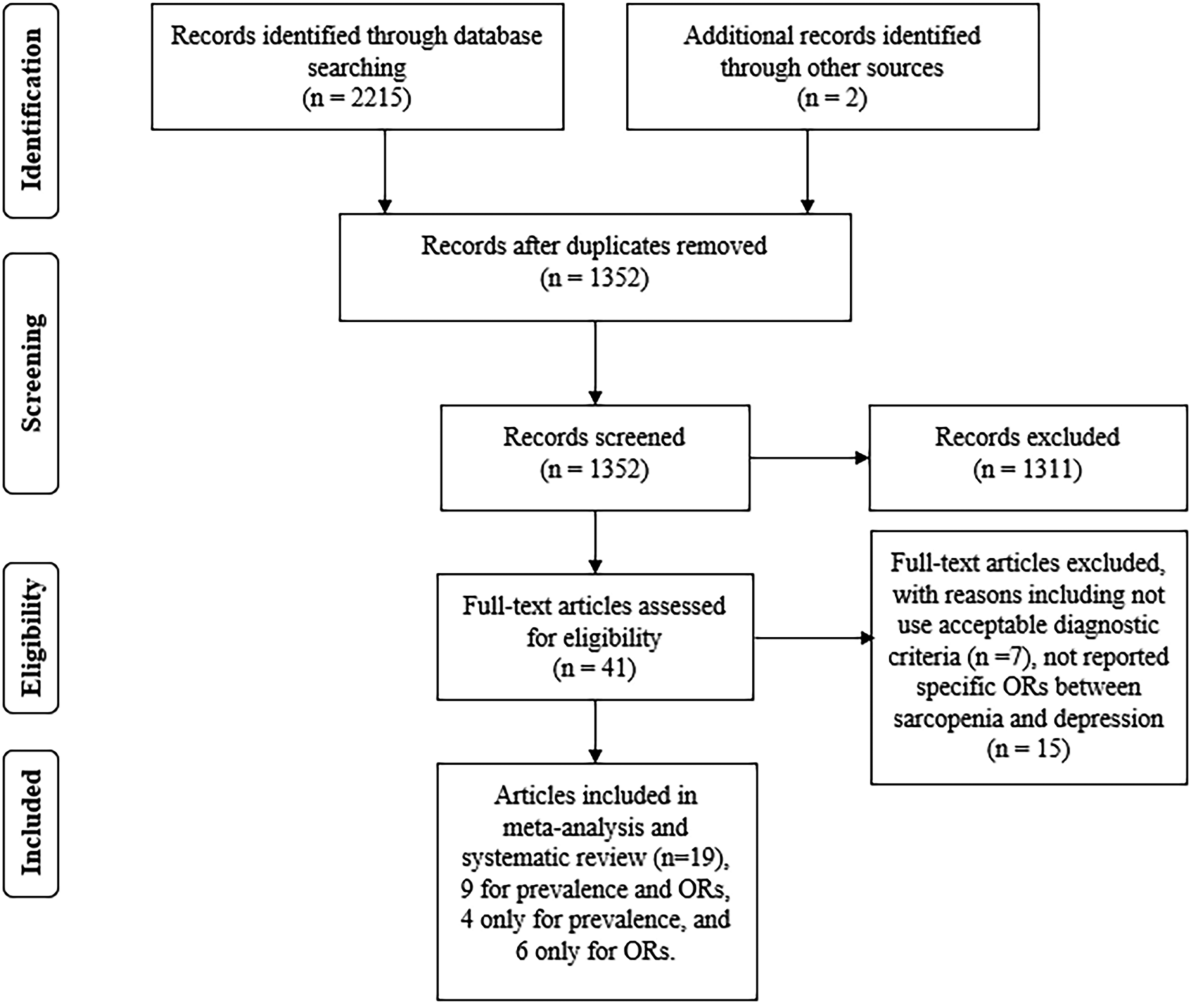
Preferred Reporting Items for Systematic Reviews and Meta‐Analyses (PRISMA) flow diagram for the study selection process. OR, odds ratio.

In all, 2215 articles were collated from the database search. After removing duplicates, 1352 titles and abstracts were screened. Forty‐one relevant studies were submitted for full‐text screening, and 22 of these were excluded. The reasons for excluding some articles after full‐text screening are shown in the flow chart and *Table* *S1*. Ultimately, 19 publications^21^, ^22^, ^28^, ^29^, ^30^, ^31^, ^32^, ^33^, ^34^, ^35^, ^36^, ^37^, ^38^, ^39^, ^40^, ^41^, ^42^, ^43^, ^44^ met the inclusion criteria for review: nine^28^, ^29^, ^34^, ^35^, ^36^, ^37^, ^39^, ^40^, ^43^ investigated both prevalence and ORs, four^21^, ^33^, ^41^, ^42^ involved prevalence only, and six^21^, ^22^, ^30^, ^31^, ^32^, ^38^ reported ORs only. *Figure* *1* is a flowchart of the literature selection process.

### Study characteristics

The characteristics of the included studies investigating prevalence are summarized in *Table* *1*. The 13 studies enrolled 1476 individuals; the mean age of the study participants ranged from 61 to 87 years. One study^29^ was separated according to sex, and one^40^ involved two separate populations (sarcopenia with obesity and sarcopenia without obesity). The characteristics of the studies reporting ORs are summarized in *Table* *2*. The 15 studies enrolled 16 869 individuals. The mean age of the study participants ranged from 61 to 82.7 years. Two studies^29^, ^39^ were separated according to sex, one study^40^ involved two separate populations (sarcopenia with obesity and sarcopenia without obesity).

**Table 1:** Characteristics of studies included in the meta‐analysis for prevalence of depression in sarcopenia

First author and year	Country and region	Study region	Study site	Study design	BMI (mean)	Sample size	No. of depression	Prevalence	Mean age (years)	Sarcopenia diagnosis standard	Criteria (assessment method to detect sarcopenia)	Depression diagnosis standard	Cut‐off value for depression
Endo (2021)	Japan	Asia	Community	Cross‐sectional	20.1	30	12	0.4	80	AWGS 2014	LMM (BIA) + LMS (HGS) + LPP (5mGS)	SDS	40
Kitamura (2021)	Japan	Asia	Community	Cross‐sectional	21.4	105	31	0.295	78	AWGS (2019)	LMM (BIA) + LMS (HGS) + LPP (5mGS)	GDS‐15	5
Kitamura (2021)	Japan	Asia	Community	Cross‐sectional	21.4	156	66	0.425	78	AWGS (2019)	LMM (BIA) + LMS (HGS) + LPP (5mGS)	GDS‐15	5
Olgun Yazar (2019)	Turkey	Asia	Community	Cross‐sectional	27.5	50	32	0.64	75	EWGSOP (2010)	LMM (BIA) + LMS (HGS) + LPP (4mGS)	GDS	11
Kilavuz (2018)	Turkey	Asia	Community	Cross‐sectional	Unknown	40	13	0.325	72	EWGSOP (2010)	LMM (MC) + LMS (HGS) + LPP (6mGS)	GDS‐15	5
Szlejf (2018)	Brazil	America	Community	Cross‐sectional	27	114	10	0.087	61	FNIH Sarcopenia Project criteria	LMM (BIA) + LMS (HGS)	B‐CIS‐R	Unknown
Hayashi (2019)	Japan	Asia	Community	Cross‐sectional	21	41	18	0.439	72.5	AWGS (2014)	LMM (BIA) + LMS (HGS) + LPP (5mGS)	GDS‐15	6
Wang (2018)	China	Asia	Community	Cross‐sectional	23.9	61	11	0.18	69	AWGS (2014)	LMM (BIA) + LMS (HGS) + LPP (6mGS)	GDS‐15	5
Sugimoto (2016)	Japan	Asia	Clinic	Cross‐sectional	19.6	88	36	0.409	80	AWGS (2014)	LMM (BIA) + LMS (HGS) + LPP (TUG)	GDS‐15	6
Ishii (2016)	Japan	Asia	Community	Cross‐sectional	Unknown	64	17	0.266	77	EWGSOP (2010)	LMM (BIA) + LMS (HGS) + LPP (5mGS)	GDS‐15	6
Ishii (2016)	Japan	Asia	Community	Cross‐sectional	Unknown	236	26	0.11	77	EWGSOP (2010)	LMM (BIA) + LMS (HGS) + LPP (5mGS)	GDS‐15	6
Huang (2015)	Taiwan, China	Asia	Community	Cross‐sectional	24.7	50	4	0.08	77	AWGS (2014)	LMM (DEXA) + LMS (HGS) + LPP (6mGS)	CES‐D	16
Alexandre (2014)	Brazil	America	Community	Cross‐sectional	21	266	36	0.135	70	EWGSOP (2010)	LMM (DEXA) + LMS (HGS) + LPP (2.4mGS)	GDS‐15	6
Hsu (2014)	Taiwan, China	Asia	Community	Cross‐sectional	20.9	109	32	0.298	84	EWGSOP (2010)	LMM (BIA) + LMS (HGS) + LPP (6mGS)	GDS‐15	6
Landi (2012)	Italy	Europe	Community	Cross‐sectional	23.8	66	20	0.3	87	EWGSOP (2010)	LMM (MC) + LMS (HGS) + LPP (4mGS)	DSM	unknown

Abbreviations: AWGS, Asian Working Group for Sarcopenia; B‐CIS‐R, Brazilian version of the Clinical Interview Scheduled Revised; BIA, bioelectrical impedance analysis; CES‐D, Center for Epidemiologic Studies Depression Scale; DSM, Diagnostic and Statistical Manual of Mental Disorders; DXA, dual‐energy X‐ray absorptiometry; EWGSOP, European Working Group on Sarcopenia in Older People; FNIH, Foundation for the National Institutes of Health; GDS, Geriatric Depression Scale; GS: gait speed; HGS, handgrip strength; LMM, lower muscle mass; LMS, lower muscle strength; LPP, lower physical performance; MC, muscle circumference; TUG, timed up and go test; SDS, Self‐rating Depression Scale.

**Table 2:** Characteristics of studies included in the meta‐analysis for ORs between sarcopenia and depression

First author and year	Country and region	Study region	Study site	Study design	Sample size	Mean age (years)	BMI (mean)	OR (95% CI)	OR (95% CI) adjusted	Adjustment factors	Sarcopenia diagnosis standard	Criteria (assessment method to detect sarcopenia)	Depression diagnosis standard	Cut‐off value for depression
Endo (2021)	Japan	Asia	Community	Cross‐sectional	155	80	22.8		1.05 (0.99–1.11)	Age, sex, the number of falls within 1 year, and chronic diseases (hypertension, dyslipidaemia, diabetes, cardio‐cerebrovascular disease)	AWGS 2014	LMM (BIA) + LMS (HGS) + LPP (5mGS)	SDS	40
Kitamura (2021)	Japan	Asia	Community	Cross‐sectional	917	78	23.4		1.2 (0.7–2.1)	Age, sex, study area, FMI, diabetes, history of stroke, anaemia, hypoalbuminaemia, current smoking, cognitive impairment, depressed mood, and hospitalization within the past year study area, fat mass index	AWGS 2019	LMM (BIA) + LMS (HGS) + LPP (5mGS)	GDS	5
Kitamura (2021)	Japan	Asia	Community	Cross‐sectional	934	78	23		2.4 (1.6–3.6)	Age, sex, study area, FMI, diabetes, history of stroke, anaemia, hypoalbuminaemia, current smoking, cognitive impairment, depressed mood, and hospitalization within the past year	AWGS 2019	LMM (BIA) + LMS (HGS) + LPP (5mGS)	GDS	5
Yuenyongchaiwat (2021)	Thailand	Asia	Hospital	Cross‐sectional	104	60	23.48		3.229 (1.139–9.157)	Age, sex, BMI, history of DM, duration of haemodialysis, levels of physical activity	AWGS 2019	LMM (BIA) + LMS (HGS) + LPP (6mGS)	BDI‐II Cut‐off value for depression: unknown
Fábrega‐Cuadros (2020)	Spain	Europe	Community	Cross‐sectional	304	72	29.14		1.10 (1.02–1.19)	Age, sex, low physical activity level, fatigue, short sleep duration	EWGSOP 2019	LMM (BIA) + LMS (HGS)	HADS Cut‐off value for depression: unknown
Yuenyongchaiwat (2020)	Thailand	Asia	Community	Cross‐sectional	330	67	25.55	2.335 (1.224–4.453)	2.089 (1.057–4.130)	Age, sex, and educational levels	AWGS 2014	LMM (BIA) + LMS (HGS) + LPP (6mGS)	GDS	12
Kilavuz (2018)	Turkey	Asia	Community	Cross‐sectional	861	72.2	Unknown		2.55 (1.11–5.88)	Age, gender, education, marital status; the perception of the economic situation; living position	EWGSOP 2010	LMM (MC) + LMS (HGS) + LPP (6mGS)	GDS	5
Szlejf (2018)	Brazil	America	Community	Cross‐sectional	5927	61	27	2.3 (1.19–4.46)	2.23 (1.11–4.48)	Age, sex, race, education, diabetes mellitus, hypertension, coronary artery disease, stroke, thyroid function status, smoking status, alcohol consumption, and leisure‐time physical activity	FNIH Sarcopenia Project criteria	LMM (BIA) + LMS (HGS)	B‐CIS‐R	unknown
Hayashi (2019)	Japan	Asia	Community	Cross‐sectional	432	72.5	22.9	2.31 (1.2–4.46)	2.38 (1.18–4.81)	Age, sex, educational history, body mass index, medical condition (hypertension, heart disease, diabetes mellitus),C‐reactive protein (CRP), interleukin‐6 (IL‐6) and physical activity (light, moderate–vigorous).	AWGS 2014	LMM (BIA) + LMS (HGS) + LPP (5mGS)	GDS	6
Wang (2018)	China	Asia	Community	Cross‐sectional	865	69	23.9	2.73 (1.35–5.11)	2.45 (1.12–5.34)	Age, gender, smoking status, alcohol drinking status, physical activity, cognitive impairment, and body fat percentage.	AWGS 2014	LMM (BIA) + LMS (HGS) + LPP (6mGS)	GDS‐15	5
Lee (2018)	Korea	Asia	Community	Cross‐sectional	201	74.3	25	5.493 (1.854–16.27)	5.448 (1.063–27.92)	Age, BMI, lean body mass, and education	AWGS 2014	LMM (DEXA) + LMS (HGS) + LPP (6mGS)	CES‐D	16
Patino‐Hernandez (2017)	USA	America	Community	Cross‐sectional	1509	76	Unknown	1.25 (0.81–1.94)	0.82 (0.5–1.36)	Sex, age, years of school, living with a partner, smokers, comorbidities, MMSE cognitive impairment, falls in the last 12 months, and unintended loss of weight.	EWGSOP 2010	LMM (MC) + LMS (HGS) + LPP (2.4mGS)	GDS‐15	6
Sugimoto (2016)	Japan	Asia	Clinic	Cross‐sectional	139	77	21.8		2.11 (0.90–4.93)	Age, education, Mini‐Mental State Examination, vitality index, depressive mood, body mass index, 25 (OH)D, serum albumin, eGFR, smoking status (only men), drinking status and No. of comorbidities; number of comorbidities (diabetes mellitus, hypertension, stroke, cardiac disease, cancer and pulmonary disease).	AWGS 2014	LMM (BIA) + LMS (HGS) + LPP (TUG)	GDS‐15	6
Sugimoto (2016)	Japan	Asia	Clinic	Cross‐sectional	279	77	21.8		1.24 (0.66–2.32)	Age, education, Mini‐Mental State Examination, vitality index, depressive mood, body mass index, 25(OH)D, serum albumin, eGFR, smoking status (only men), drinking status and no. of comorbidities; number of comorbidities (diabetes mellitus, hypertension, stroke, cardiac disease, cancer and pulmonary disease).	AWGS 2014	LMM (BIA) + LMS (HGS) + LPP (TUG)	GDS‐15	6
Ishii (2016)	Japan	Asia	Community	Cross‐sectional	1732	77	Unknown		2.79 (1.43–5.43)	Age, sex, food intake, poor sleep, physical activity, education level, social isolation, living alone, neighbourhood ties, chronic comorbidity burden, use of antidepressant, and use of statin	EWGSOP 2010	LMM (BIA) + LMS (HGS) + LPP (5mGS)	GDS‐15	6
Ishii (2016)	Japan	Asia	Community	Cross‐sectional	1732	77	Unknown		0.93 (0.55–1.60)	Age, sex, food intake, poor sleep, physical activity, education level, social isolation, living alone, neighbourhood ties, chronic comorbidity burden, use of antidepressant, and use of statin	EWGSOP 2010	LMM (BIA) + LMS (HGS) + LPP (5mGS)	GDS‐15	6
Hsu (2014)	Taiwan, China	Asia	Community	Cross‐sectional	353	82.7	23	2.55 (1.41–4.6)	2.25 (1.03–4.89)	Age, body mass index, physical function, chronic obstructive pulmonary disease, cognitive impairment	EWGSOP 2010	LMM (BIA) + LMS (HGS) + LPP (6mGS)	GDS‐15	6
Kim (2013)	Korea	Asia	Hospital	Cross‐sectional	95	63.9	22.3	8.75 (2.74–27.9)	6.87 (2.06–22.96)	Age, gender, BMI, diabetes	EWGSOP 2010	LMM (BIA) + LMS (HGS)	BDI‐II	16

Abbreviations: AWGS, Asian Working Group for Sarcopenia; B‐CIS‐R, Brazilian version of the Clinical Interview Scheduled Revised; BDI‐II, Beck Depression Inventory‐II; BIA, bioelectrical impedance analysis; BMI, body mass index; CES‐D, Center for Epidemiologic Studies Depression Scale; DXA, dual‐energy X‐ray absorptiometry; EWGSOP, European Working Group on Sarcopenia in Older People; FNIH, Foundation for the National Institutes of Health; GDS, Geriatric Depression Scale; GS, gait speed; HADS, Hospital Anxiety and Depression Scale; HGS, handgrip strength; LMM, lower muscle mass; LMS, lower muscle strength; LPP, lower physical performance; MC, muscle circumference; SDS, Self‐rating Depression Scale; TUG, timed up and go test.

Most of the included studies were conducted in the community and Asia, and all studies adopted a cross‐sectional design. Almost all studies defined sarcopenia as the presence of LMM, LMS, and low physical fitness (LPP). To diagnose depression, two studies used the Geriatric Depression Scale (GDS), and nine used the GDS, 15‐item version (GDS‐15), two used the Center for Epidemiological Studies Depression Scale (CES‐D), two used the Beck Depression Inventory‐II, one study used the Zung Self‐rating Depression Scale (SDS), one used the Diagnostic and Statistical Manual of Mental Disorders, and one used the Hospital Anxiety and Depression Scale.

### Diagnostic method for sarcopenia

The details of the diagnostic criteria and cut‐off points for sarcopenia in each study are listed in *Table* *3*. Sixteen studies defined sarcopenia by combining LMM, LMS, and LPP, while three used LMM and LMS. Different measurement methods and cut‐off values were utilized to confirm LMM, LMS, and LPP in these studies. Muscle mass was assessed using bioelectrical impedance analysis (13 studies), dual‐energy X‐ray absorptiometry (three studies), calf circumference (two studies), and mid‐arm muscle circumference (one study). Muscle strength was detected using handgrip dynamometry, and physical performance was detected using the 2.4, 4, 5, and 6 m walk test, gait speed, or timed up‐and‐go test.

**Table 3:** The details of diagnosis criteria and cut‐off points of each study

LMM		References
BIA	AWGS 2019^45^ or 2014^46^: SMI < 7.0 kg/m^2^ for men and <5.7 kg/m^2^ for women	Endo (2021), Kitamura (2021), Yuenyongchaiwat (2021), Hayashi (2019), Wang (2018), Yuenyongchaiwat (2020), Sugimoto (2016)
	EWGSOP 2010: SMI < 10.52 kg/m^2^ for men and <8.87 kg/m^2^ for women	Olgun Yazar (2019)
	EWGSOP 2010^47^: SMI < 7.0 kg/m^2^ for men and <5.8 kg/m^2^ for women	Ishii (2016)
	FNIH^48^ ALM/BMI: <0.789 for men and for <0.512 for women	Szlejf (2018)
	EWGSOP 2010 Chien *et al*. (2008)^49^: SMI < 8.87 kg/m^2^ for men and <6.42 kg/m^2^ for women	Ying‐Hsin Hsu (2014), Fábrega‐Cuadros (2020)
	EWGSOP 2010 Chien *et al*. (2008)^5^: Lean Tissue Index (LTI) of 2 standard deviations (SD) or more below the normal gender‐specific means for young persons	Kim (2013)
DEXA	AWGS 2014^46^: SMI < 7.0 kg/m^2^ for men and <5.4 kg/m^2^ for women	Huang (2015)
	EWGSOP 2010 Newman *et al*. (2003),^50^ Delmonico MJ *et al*. (2007)^51^: SMI < 8.90 kg/m^2^ for men and <6.37 kg/m^2^ for women	Alexandre (2014)
	AWGS 2014: SMI < 5.4 kg/m^2^	Lee (2018)
MC	EWGSOP 2010: calf circumference <31 cm	Kilavuz (2018), Patino‐Hernandez (2017)
	EWGSOP 2010^52^: mid‐arm muscle circumference <21.1 cm for men and <19.2 cm for women	Landi (2012)
LMS		
HGS	AWGS 2019^45^: < 28 kg for men and < 18 kg for women	Kitamura (2021), Yuenyongchaiwat (2021)
	EWGSOP 2010^53^: < 30 kg for men and < 20 kg for women	Olgun Yazar (2019), Kilavuz (2018), Ishii (2016), Alexandre (2014), Landi (2012), Kim (2013)
	FNIH^54^ < 26 kg for men and < 16 kg for women	Szlejf (2018)
	AWGS 2014^46^: < 26 kg for men and < 18 kg for women	Endo (2021), Hayashi (2019), Wang (2018), Sugimoto (2016), Huang (2015), Yuenyongchaiwat (2020)
	EWGSOP 2010: < 24 kg/m^2^ for men and < 14 kg/m^2^ for women	Patino‐Hernandez (2017)
	EWGSOP 2010^55^, ^56^: < 22.5 kg	Hsu (2014)
	EWGSOP 2019^57^: < 27 kg for men and < 16 kg for women	Fábrega‐Cuadros (2020)
	AWGS 2014: < 18 kg	Lee (2018)
LPP		
2.4mGS	EWGSOP 2010: GS < 0.8 m/s	Alexandre (2014)
2.4mGS	EWGSOP 2010: GS < 0.48 m/s	Patino‐Hernandez (2017)
4mGS	EWGSOP 2010: GS < 0.8 m/s	Olgun Yazar (2019), Landi (2012)
5mGS	AWGS 2019: GS < 1.0 m/s	Kitamura (2021)
5mGS	AWGS 2014: GS < 0.8 m/s	Hayashi (2019), Endo (2021)
6mGS	EWGSOP 2010: GS < 1.0 m/s	Kilavuz (2018)
6mGS	AWGS 2019: GS < 1.0 m/s	Yuenyongchaiwat (2021)
6mGS	AWGS 2014: GS < 0.8 m/s	Wang (2018), Huang (2015), Yuenyongchaiwat (2020), Lee (2018)
6mGS	EWGSOP 2010: GS < 0.8 m/s	Hsu (2014)
5mGS	EWGSOP 2010: GS < 1.26 m/s	Ishii (2016)
Timed up and go test	AWGS 2014: TUG time > 13.56 s	Sugimoto (2016)

Abbreviations: AWGS, Asian Working Group for Sarcopenia; BIA, bioelectrical impedance analysis; EWGSOP, European Working Group on Sarcopenia in Older People; FNIH, Foundation for the National Institutes of Health; GS, gait speed; HGS, handgrip strength; LMM, lower muscle mass; LMS, lower muscle strength; LPP, lower physical performance; MC, muscle circumference.

### Meta‐analysis results

#### Overall results of prevalence

**Figure 2:**
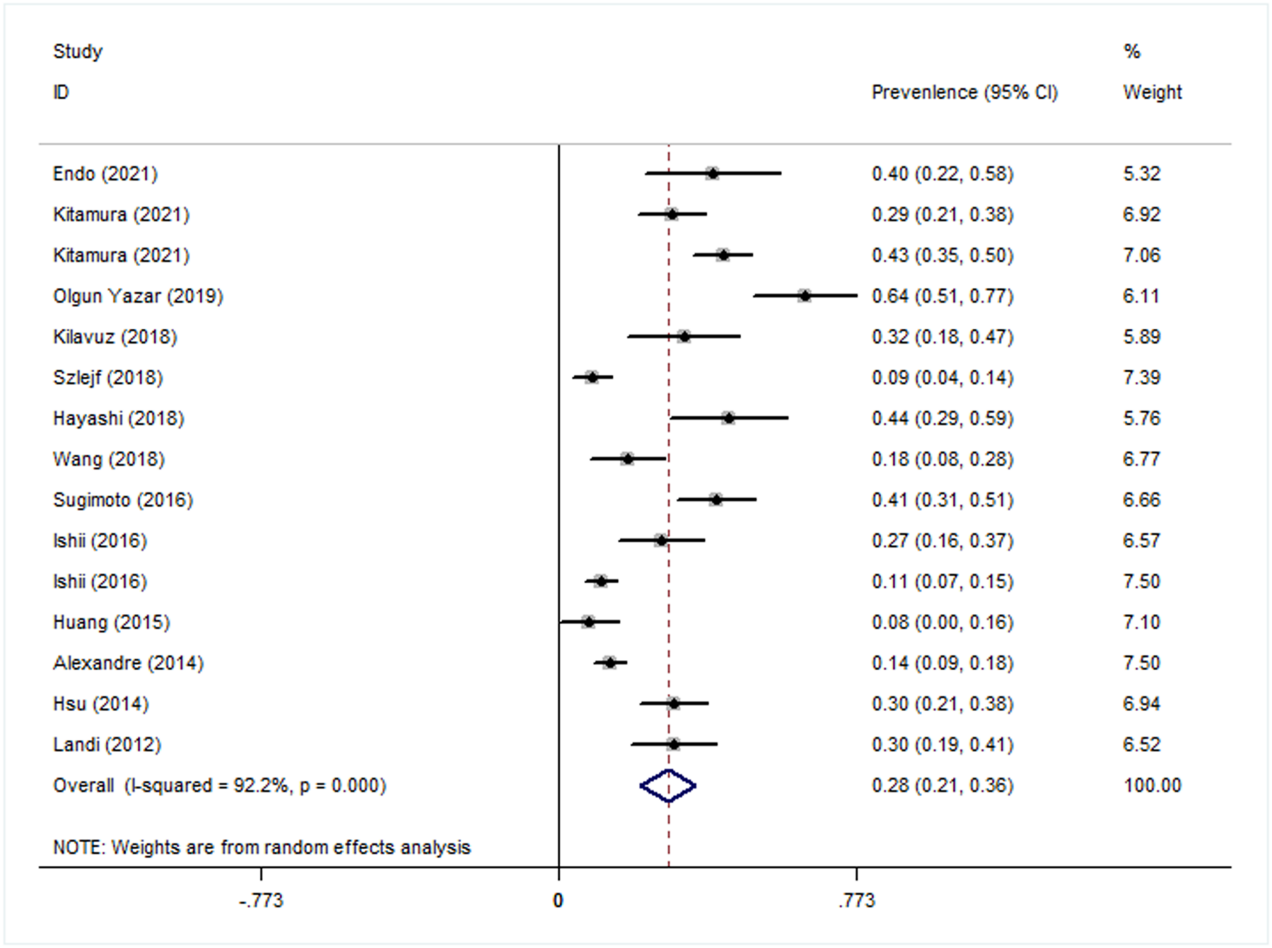
Forest plot of prevalence of depression in sarcopenia. CI, confidence interval, OR, odds ratio.

There were 1476 cases of sarcopenia and 364 of depression in the selected studies; the patients' mean age was 75.5 years. The meta‐analysis showed that the overall prevalence of depression in patients with sarcopenia was 0.28 (95% CI: 0.21–0.36). (*Figure* *2*) A significant heterogeneity was noted (*P* < 0.001; *I* ^2^ = 92.2%).

### Subgroup analyses

The subgroup analysis showed that the prevalence of depression in patients with sarcopenia was 0.31 (95% CI: 0.20–0.43) in the group diagnosed with sarcopenia according to the Asian Working Group for Sarcopenia (AWGS), 0.29 (95% CI: 0.18–0.39) in the group diagnosed according to the European Working Group on Sarcopenia in Older People (EWGSOP), 0.32 (95% CI: 0.22–0.41) in the group diagnosed using bioelectrical impedance analysis (BIA), 0.28 (95% CI 0.20–0.36) in the group diagnosed using the GDS‐15, 0.36 (95% CI: −0.18–0.90) in those with overweight, 0.32 (95% CI: 0.22–0.41) in Asia, and 0.27 (95% CI: 0.20–0.35) in the community (*Table* *4*).

**Table 4:** The results of subgroup analysis in prevalence of depression in sarcopenia

Outcomes	Numbers of studies	Meta‐analysis results	*I* ^2^ (%)	*P*
Diagnostic criteria for sarcopenia				
AWGS	6	0.31 (0.20–0.43)	89.4	<0.001
EWGSOP	6	0.29 (0.18–0.39)	92.5	<0.001
FNIH	1	0.09 (0.04–0.14)	/	/
Measurement method for LMM				
BIA	9	0.32 (0.22–0.41)	93.4	<0.001
DEXA	2	0.12 (0.07–0.17)	36.8	0.208
Calf circumference	1	0.32 (0.18–0.47)	/	/
Mid‐arm muscle circumference	1	0.30 (0.19–0.41)	/	/
Measurement method for depression				
GDS‐15	8	0.28 (0.20–0.36)	91.1	<0.001
GDS	1	0.64 (0.51–0.77)	/	/
CISR‐B	1	0.09 (0.04–0.14)	/	/
CES‐D	1	0.08 (0.00–0.16)	/	/
SDS	1	0.40 (0.22–0.58)	/	/
DSM	1	0.30 (0.19–0.41)	/	/
BMI				
Overweight	2	0.36 (−0.18–0.90)	98.3	<0.001
Normal	9	0.29 (0.20–0.38)	90.0	<0.001
Unknown	2	0.22 (0.08–0.36)	85.3	0.001
Ethnicity				
Asia	10	0.32 (0.22–0.41)	92.2	<0.001
America	2	0.11 (0.07–0.16)	50.7	0.154
Europe	1	0.30 (0.19–0.41)	/	/
Site				
Community	12	0.27 (0.20–0.35)	91.9	<0.001
Clinic	1	0.41 (0.31–0.51)	/	/

Abbreviations: BIA, bioelectrical impedance analysis; DEXA, dual‐energy X‐ray absorptiometry; GDS, Geriatric Depression Scale; GDS‐15, 15‐item Geriatric Depression Scale; CES‐D, Center for Epidemiologic Studies Depression Scale; SDS, Self‐rating Depression Scale; CISR‐B, Brazilian version of the Clinical Interview Scheduled Revised; DSM, Diagnostic and Statistical Manual of Mental Disorders.

### Sensitivity analysis

Sugimoto *et al*.^39^ conducted a study in patients with amnestic mild cognitive impairment or Alzheimer's disease. Hsu^43^ recruited veterans for their study involving all male participants living in the same retirement community and sharing a similar life course from China to Taiwan during the Chinese Civil War. Therefore, we performed a sensitivity analysis excluding those two studies to investigate the impact of these studies on the aggregate results. The sensitivity analysis found that the combined prevalence did not change substantially, indicating that our meta‐analysis was stable (*Figure* *S1*).

### Overall results of odds ratios

**Figure 3:**
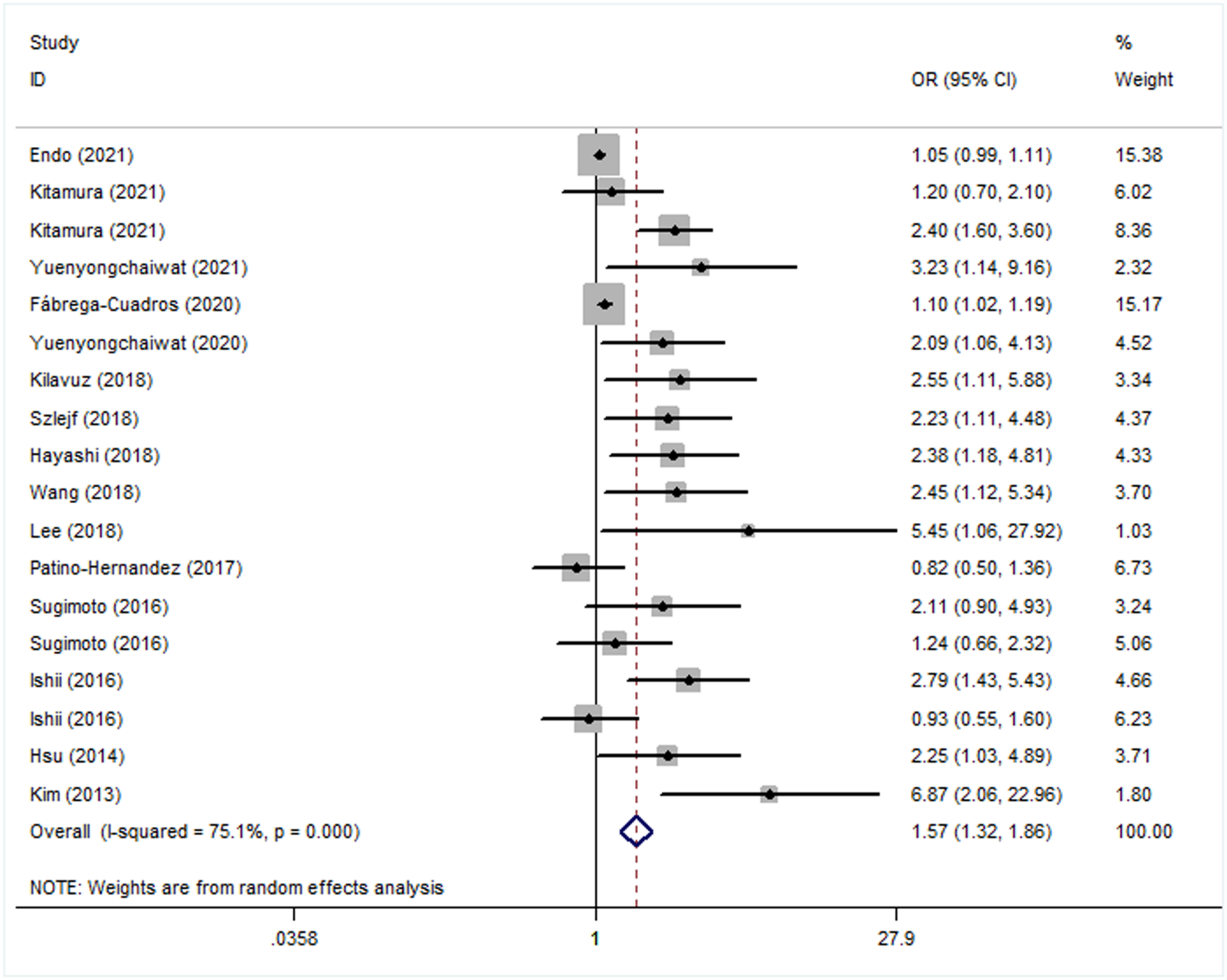
Forest plot of the adjusted odds ratios (ORs) between sarcopenia and depression. CI, confidence interval

There were 16 869 subjects, with a mean age of 73 years. The overall adjusted OR between sarcopenia and depression was 1.57 (95% CI: 1.32–1.86; *Figure* *3*). Moderate heterogeneity in both adjusted ORs (*P* < 0.001; *I* ^2^ = 75.1%) was noted across the studies.

### Subgroup analyses

Results of the subgroup analysis showed that the adjusted OR between depression and sarcopenia was 1.85 (95% CI: 1.30–2.63) in the group diagnosed with sarcopenia according to the AWGS, 1.60 (95% CI: 1.07, 2.40) in those diagnosed according to the EWGSOP, 1.58 (95% CI: 1.32, 1.89) in the group diagnosed using BIA, 1.54 (95% CI: 1.04–2.29) in the group diagnosed using GDS‐15, 1.81 (95% CI: 1.01, 3.25) in those with overweight, 1.97 (95% CI: 1.45, 2.67) in Asia, and 1.47 (95% CI: 1.24, 1.75) in the community (*Table* *5*).

**Table 5:** The results of subgroup analysis for ORs between sarcopenia and depression

Outcomes	Numbers of studies	Meta‐analysis results	*I* ^2^ (%)	*P*
Diagnostic criteria for sarcopenia				
AWGS	8	1.85 (1.30–2.63)	77.0	<0.001
EWGSOP	6	1.60 (1.07–2.40)	75.9	<0.001
FNIH	1	2.23 (1.11–4.48)	/	/
Measurement method for LMM				
BIA	12	1.58 (1.32–1.89)	76.4	<0.001
DEXA	1	5.45 (1.06–27.92)	/	/
Calf circumference	2	1.37 (0.45–4.16)	80.9	0.022
Measurement method for depression				
GDS‐15	5	1.54 (1.04–2.29)	60.5	0.019
GDS	4	2.02 (1.52–2.69)	14.3	0.323
CISR‐B	1	2.23 (1.11–4.48)	/	/
CES‐D	1	5.45 (1.06–27.92)	/	/
SDS	1	1.05 (0.99–1.11)	/	/
HADS	1	1.10 (1.02–1.19)	/	/
BDI‐II	2	4.46 (2.03–9.81)	0	0.353
BMI				
Overweight	4	1.81 (1.01–3.25)	72.1	0.013
Normal	8	1.95 (1.34–2.82)	79.6	<0.001
Unknown	3	1.45 (0.78–2.71)	75.6	0.006
Ethnicity				
Asia	12	1.97 (1.45–2.67)	77.7	<0.001
America	2	1.31 (0.49–3.49)	80.8	0.022
Europe	1	1.10 (1.02–1.19)	/	/
Site				
Community	12	1.47 (1.24–1.75)	75.4	<0.001
Clinic	1	1.50 (0.90–2.48)	/	/
Hospital	2	4.46 (2.03–9.81)	0	0.353

Abbreviations: BIA, bioelectrical impedance analysis; CES‐D, Center for Epidemiologic Studies Depression Scale; DEXA, dual‐energy X‐ray absorptiometry; GDS, Geriatric Depression Scale; GDS‐15, 15‐item Geriatric Depression Scale; SDS, Self‐rating Depression Scale; CISR‐B, Brazilian version of the Clinical Interview Scheduled Revised; HADS, Hospital Anxiety and Depression Scale.

### Sensitivity analysis

Similar to the sensitivity analysis for prevalence, a sensitivity analysis was conducted excluding four of the OR studies: Yuenyongchaiwat,^30^ which was conducted in patients with chronic renal failure undergoing haemodialysis; Sugimoto,^39^ which was carried out in patients with amnestic mild cognitive impairment or Alzheimer's disease; Hsu,^43^ which involved veterans; Kim,^21^ which was performed in patients with end‐stage renal disease. The sensitivity analysis found that the combined adjusted ORs did not change substantially, indicating that our meta‐analysis was stable (*Figure* *S2*).

### Overall assessment of evidence quality

The overall quality of each study was relatively high (*Table* *S2*).

## Discussion

### Summary of main results

The present study aimed to integrate the latest evidence in a study of the prevalence of depression in patients with sarcopenia and to assess the association between sarcopenia and depression. The results showed that the prevalence of depression was high in patients with sarcopenia and that there was a positive correlation between sarcopenia and depression. This relationship was not affected by adjustment for related covariates.

### Subgroup analysis according to different muscle mass measurement methods

In the meta‐analysis conducted in subgroups according to ethnicity, research locations, muscle mass and depression measurement methods, diagnostic criteria for sarcopenia, and BMI, the differences between the subgroups were also significant. Dual X‐ray absorptiometry (DXA) diagnosis resulted in a lower prevalence of depression than BIA, and the correlation between depression and sarcopenia was more marked using the former. This showed that different sarcopenia diagnostic methods significantly affect the prevalence and ORs of depression in patients with sarcopenia; however, because most of the included studies involved healthy elderly people in the community, almost all were conducted using BIA to measure muscle mass. Thus, more research using DXA is needed to confirm the impact of different measurement methods. DXA and BIA are two commonly used techniques for evaluating body composition. DXA can quickly and non‐invasively evaluate fat mass, fat‐free mass, and bone mineral density, but it requires specialized radiological equipment and is expensive.^58^ BIA is regarded as a portable alternative to DXA and is more widely used in clinical practice.^59^, ^60^ A retrospective study comparing DXA and BIA found that the BIA and DXA methods are interchangeable at the population level.^61^ So BIA is recommended for future sarcopenia research because it is non‐invasive, economical, convenient and easier to popularize.

### Subgroup analysis according to different depression screening tools

Similarly, differences in the depression scales used also affected the prevalence of depression in patients with sarcopenia, as well as the correlation between the two; moreover, the difference between subgroups was significant. However, most studies used GDS or GDS‐15, with fewer studies using other scales. The prevalence of depression was higher when diagnosed using the GDS than when using the GDS‐15, and the correlation between depression and sarcopenia was more marked using the former. The meta‐analysis showed that the pooled sensitivity and specificity of GDS were 82% and 76%, respectively, with a near higher diagnostic accuracy (area under the curve = 0.85). GDS‐15 had pooled sensitivity and specificity values of 86% and 79%, respectively, with higher diagnostic accuracy (area under the curve = 0.90). The diagnostic performance of the GDS‐15 was much better than that of the GDS.^62^ So GDS‐15 is recommended for future depression screening tool.

### Reasons for the heterogeneity

Although subgroup and sensitivity analyses were performed, heterogeneity was still very large in the meta‐analysis, perhaps for the following reasons: First, although sarcopenia was diagnosed based on LMM and LMS in the included studies, and most measured muscle function, the degree of sarcopenia was not graded, so the severity of sarcopenia may not have been consistent across studies. Second, most of the included studies used the GDS or GDS‐15 to determine depressive symptoms. The GDS is an effective measurement method for assessing depressive symptoms, satisfactory psychometric properties and broad clinical use to evaluate elderly people in various environments. However, it is only a depression screening tool, not a diagnostic tool, and depressive symptoms associated with GDS may be related to physical illness.^63^ In addition, the severity of depression in the included study subjects was not the same; the diagnosis of depression used questionnaires (such as GDS, BDI‐II, and SDS), so self‐report bias and recall bias were inevitable. This may have affected the prevalence of sarcopenia and depressive symptoms to varying degrees. Third, although the ORs were adjusted for demographic data, including age, sex, BMI, education, and many confounding factors, such as disability, weakness, physical activity, and sex hormones, may have affected the association between sarcopenia and depression. The present study could not unify all confounding factors. Fourth, the subjects in the included studies were older people; most of them had multiple comorbidities, each of which affected the others. Higher comorbidity index was associated with higher prevalence of depression. There were also differences in comorbidities among the included studies. Finally, differences in race, region, and quality control of the research process between studies may lead to greater heterogeneity.

### Mechanism basis

Recently, researchers have recognized the relationship between depressive symptoms and sarcopenia; there are multiple mechanisms that interact with both sarcopenia and depression, including neurotrophins, oxidative stress, inflammation, and the regulation of lifestyle behaviours. Brain‐derived neurotrophic factor is one of the most explored biomarkers of depression; it is produced in various tissues, including skeletal muscle, acts on neurons in the central nervous and peripheral nervous systems, and drives hippocampal neurogenesis, the hippocampus being a key area of the brain that is related to mental illness.^64^, ^65^, ^66^ Age‐related chronic low‐grade inflammation is an important cause of both sarcopenia and depression. It is characterized by elevated levels of tumour necrosis factor‐alpha, C‐reactive protein, and interleukin‐6. These inflammatory cytokines can increase the interstitial concentrations of norepinephrine, dopamine, serotonin, and their metabolites in the hypothalamus and hippocampus, which are related to mental illness. Sarcopenia may adversely affect mental function through metabolic and endocrine mechanisms. Disability and insufficient physical activity due to decreased muscle strength and muscle mass may cause depression.^67^ Conversely, depression leads to a decline in social activity and can lead to sarcopenia. Our findings are consistent with this mechanism, indicating that there is indeed a correlation between sarcopenia and depression, but almost all selected articles had a cross‐sectional design and could not confirm causality between the analysed factors. Chen *et al*.^68^ performed a year‐long cohort study and showed that sarcopenia is an independent risk factor for depressive symptoms, but their study had many limitations. In particular, their study population may not have been comprehensive enough, as all participants were relatively healthy, and the follow‐up time was short. Therefore, a rigorous, large‐sample, long‐term prospective cohort study will be necessary to confirm the causal relationship between sarcopenia and depression.

### New discoveries and trends

In one study,^40^ the prevalence and OR of depression in the sarcopenia with obesity group were significantly higher than those in the sarcopenia without obesity group, indicating that obesity can increase the risk of depression. Obesity is an established risk factor for mental disorders and depression^69^; obesity and depression have mutual influence, and an activated hypothalamic–pituitary–adrenal axis and inflammation are considered common mediating pathways for depression and obesity.^70^ Because sarcopenia and obesity both promote depression, patients with both sarcopenia and obesity are at higher risk of depression than those with sarcopenia alone. However, it remains unclear whether the combination of sarcopenia and obesity has an additive or a synergistic effect. BMI is currently the most useful obesity measurement index at the population level, but the association between BMI and depression is inconclusive. Some studies have indicated that an increase in BMI can reduce the risk of depression,^71^, ^72^, ^73^, ^74^ while other studies have shown that patients with a higher BMI are more likely to suffer from depression.^75^ A large nationally representative study in the United States showed a U‐shaped relationship between the prevalence of lifetime‐diagnosed depression and BMI: people with a BMI lower than 18.5 kg/m^2^ or higher than 30 kg/m^2^ have a higher risk of depression than those with a normal BMI (18.5–24.9 kg/m^2^).^76^ This shows that both increasing and decreasing BMI can increase the risk of depression. Many previous studies have used BMI to define obesity; however, it cannot distinguish between muscle and fat, so it is not a standardized indicator to determine overweight. Lower BMI does not indicate good health, because the respective individual may have more fat; conversely, a higher BMI may not denote obesity, but it may mean more muscle mass. Previous studies have shown that the severity of sarcopenia decreases linearly with BMI, and a higher BMI may reflect better nutrition and regular physical activity, including exercise, which is conducive to increased muscle mass and helps prevent or delay sarcopenia.^77^ Therefore, it is difficult to accurately evaluate health risks using BMI to assess obesity. Muscles play an important role in human bodily functions and diseases; therefore, body composition analysis using percentage body fat to evaluate body adiposity should be used to distinguish between muscle mass and body fat mass.

In addition, Kitamura^29^ calculated the prevalence of depression in patients with sarcopenia of each sex. The findings showed that the prevalence and OR of depression in women with sarcopenia were higher than those in men with sarcopenia, perhaps because women are more likely to suffer from sarcopenia and depression than men, or because of a demographic bias. Yazar's^33^ research divided men and women into pre‐sarcopenia, sarcopenia, and severe sarcopenia groups according to the degree of the disease; the degree of sarcopenia was determined by muscle mass, muscle strength, and physical function, and the prevalence of depression was not significantly different between groups, indicating that depression may be more related to muscle mass than to muscle strength and physical function. Byeon's study^78^ calculated the association between LMM and depression. Patients with depression were classified as either those diagnosed with depression or those with depressive symptoms. The depression group consisted of patients who had been clinically diagnosed with depression, while the depressed group consisted of patients who had specific depressive symptoms in the past year. The results showed that the association between depression and low skeletal muscle mass was not significantly different between the two groups. This indicates that the specific disease state of patients with depression does not affect the correlation between depression and sarcopenia. Another report showed that increased depression severity was associated with sarcopenia,^79^ and that sarcopenia stages varied according to the severity of depression. The relationship between depression severity and the stages of sarcopenia remains unclear.

### Agreements and disagreements with other studies or reviews

Our research results are consistent with the results of a previous meta‐analysis^23^ on the correlation between sarcopenia and depression. Both studies demonstrated that sarcopenia has a significant positive association with depression. In the present meta‐analysis, only eight^21^, ^39^, ^41^, ^42^, ^43^, ^44^, ^80^, ^81^ of the 15 included studies used muscle mass combined with muscle strength to diagnose sarcopenia; six of them met our inclusion criteria and were included in our analysis, while three other studies^71^, ^78^, ^82^ diagnosed sarcopenia based on measured muscle mass only: two used the DXA and one used the BIA. Four studies^83^, ^84^, ^85^, ^86^ used decreased grip strength as a measure of sarcopenia. Older people can be classified into three stages: robust, pre‐sarcopenia, and sarcopenia; pre‐sarcopenia describes the transition stage from robust to sarcopenia^87^; it is characterized by LMM and normal muscle strength. Muscle mass, grip strength, and gait speed are the three elements used to assess sarcopenia; one study on the risk of death from disability related to sarcopenia pointed out that, in the absence of low grip strength and slow gait, LMM does not significantly increase the risk of death and accidental disability; at the same time, in the absence of LMM, low grip strength and/or slow gait do not increase the risk of all‐cause mortality.^29^ This suggests that the current major sarcopenia algorithms, EWGSOP2 and AWGS, are effective, and that sarcopenia can be diagnosed based on the presence of LMS together with LMM, according to given cut‐off points. The included studies in our analysis met the criteria and defined sarcopenia as the presence of LMM, LMS, and/or LPP, but there were no strict diagnostic criteria in the previous meta‐analysis. Moreover, few studies have evaluated the association between depression and sarcopenia defined as LMM and low muscle function and/or poor physical performance, so few met the inclusion criteria of our meta‐analysis. As a result, our subgroup analysis was limited and showed broad heterogeneity.

### Strengths and weaknesses

In the current meta‐analysis, we feel confident that our literature search was thorough and that no relevant trials were missed. We conducted trial selection, data extraction, and quality assessments to minimize bias and transcription errors. We used standard scales to evaluate the quality of the included literature, repeatedly confirmed the meta‐results, and reviewed a large number of manuscripts to study the mechanism of the results.

Although the results are encouraging, the study had several limitations. First, the meta‐analysis included only English‐language studies identified in a few databases, which may have led to the exclusion of high‐quality, non‐English studies. Second, all selected articles had cross‐sectional design, so we could not establish a causal relationship between sarcopenia and depression. Third, there was significant heterogeneity among the included studies in terms of the diagnostic methods, measurement approaches, and diagnostic thresholds; moreover, the adjusted variables differed among the retrieved studies. For these reasons, residual bias and confounding remained a possibility. Fourth, no subgroup analysis was performed based on the severity of sarcopenia and depression. In addition, limited by the inclusion criteria, the number of studies was small, the study population mainly included Asians, the locations were mostly in communities, and most studies used BIA and/or the GDS as measurement approaches, which limit global adaptability.

## Conclusion

### Implications for clinical practice

The prevalence of depression in patients with sarcopenia is higher than in the general population, and there is a significant association between sarcopenia and depression. In future clinical work, attention should be paid to screening for depression in patients with sarcopenia, and the mutual influence of sarcopenia and depression should be recognized; in the management of sarcopenia, attention should be paid to the management of depression. Such measures will control the development of the disease, reduce complications, improve quality of life, and alleviate the social and economic burden.

### Implications for research

The present systematic review has the following implications for future research: First, the current studies mainly included Asians and healthy people in the community, so the association between sarcopenia and depression should be verified in different races and in older people with comorbidities, such as diabetes, hypertension, and other common diseases. Second, a variety of measurement methods were used to diagnose sarcopenia, so unified measurement tools should be used to diagnose sarcopenia in the future. Third, sarcopenia and depression should be classified to verify the impact of disease severity on the prevalence of the other disease. Fourth, we will perform a large‐scale, prospective cohort study stratified based on the population, region, age, and sex to assess the prevalence of depression in patients with sarcopenia and the relationship between depression and sarcopenia. Finally, in addition to studying the effect of sarcopenia on depression, the effect of depression on sarcopenia must also be studied.

## Conflict of interest

The authors declare that they have no conflicts of interest.

## Support

This study was supported by grants from Chinese National Science & Technology Pillar Program(2020YFC2005600); Sichuan Science and Technology Program (2021YFS0136); 1.3.5 project for disciplines of excellence, West China Hospital, Sichuan University (ZYJC21005); 1·3·5 project for disciplines of excellence–Clinical Research Incubation Project, West China Hospital, Sichuan University(19HXFH012), National Clinical Research Center for Geriatrics, West China Hospital, Sichuan University (Z20191003).

## Supporting information


**Table S1.** The reasons for the exclusion of full‐text articles.

**Table S2.** Risk of bias of the included studies using the Newcastle–Ottawa Scale.

**Figure S1.** The sensitivity analysis for prevalence of depression in sarcopenia.

**Figure S2.** The sensitivity analysis for adjusted ORs between sarcopenia and depression.

## References

[1] Eurelings LS , van Dalen JW , Ter Riet G , Moll van Charante EP , Richard E , van Gool WA , et al. Apathy and depressive symptoms in older people and incident myocardial infarction, stroke, and mortality: a systematic review and meta‐analysis of individual participant data. Clin Epidemiol 2018;10:363–379. 29670402 10.2147/CLEP.S150915 PMC5894652

[2] Schillerstrom JE , Royall DR , Palmer RF . Depression, disability and intermediate pathways: a review of longitudinal studies in elders. J Geriatr Psychiatry Neurol 2008;21:183–197. 18838741 10.1177/0891988708320971

[3] Fleischmann A , Arensman E , Berman A , Carli V , De Leo D , Hadlaczky G , et al. Overview evidence on interventions for population suicide with an eye to identifying best‐supported strategies for LMICs. Global Mental Health (Cambridge, England) 2016;3:e5. 10.1017/gmh.2015.27 PMC5314741 28596874

[4] Heok KE , Ho R . The many faces of geriatric depression. Curr Opin Psychiatry 2008;21:540–545. 18852559 10.1097/YCO.0b013e328311cdae

[5] WHO Press, Geneva World Health Organization , 2018. Depression. Available from http://www.who.int/mediacentre/factsheets/fs369/en/ (accessed 11 April 2018).

[6] Hasin DS , Sarvet AL , Meyers JL , Saha TD , Ruan WJ , Stohl M , et al. Epidemiology of Adult DSM‐5 major depressive disorder and its specifiers in the United States. JAMA Psychiat 2018;75:336–346. 10.1001/jamapsychiatry.2017.4602 PMC5875313 29450462

[7] Ferrari AJ , Charlson FJ , Norman RE , Patten SB , Freedman G , Murray CJ , et al. Burden of depressive disorders by country, sex, age, and year: findings from the Global Burden of Disease Study 2010. PLoS Med 2013;10:e1001547. 24223526 10.1371/journal.pmed.1001547 PMC3818162

[8] Gariballa S , Alessa A . Associations between low muscle mass, blood‐borne nutritional status and mental health in older patients. BMC Nutr 2020;6:6. 32190345 10.1186/s40795-019-0330-7 PMC7066831

[9] Yuenyongchaiwat K , Buranapuntalug S , Pongpanit K , Kulchanarat C , Satdhabudha O . Anxiety and depression symptomatology related to inspiratory muscle strength and functional capacity in preoperative cardiac surgery patients: a preliminary cross‐sectional study. Indian J Psychol Med 2020;42:549–554. 33354081 10.1177/0253717620930318 PMC7735231

[10] Moon JH , Kong MH , Kim HJ . Low muscle mass and depressed mood in korean adolescents: a cross‐sectional analysis of the fourth and fifth Korea National Health and Nutrition Examination Surveys. J Korean Med Sci 2018;33:e320. 30534032 10.3346/jkms.2018.33.e320 PMC6281954

[11] Milaneschi Y , Simmons WK , van Rossum EFC , Penninx BW . Depression and obesity: evidence of shared biological mechanisms. Mol Psychiatry 2019;24:18–33. 29453413 10.1038/s41380-018-0017-5

[12] Gariballa S , Alessa A . Sarcopenia: prevalence and prognostic significance in hospitalized patients. Clin Nutr (Edinburgh, Scotland) 2013;32:772–776. 10.1016/j.clnu.2013.01.010 23395102

[13] Cruz‐Jentoft AJ , Baeyens JP , Bauer JM , Boirie Y , Cederholm T , Landi F , et al. Sarcopenia: European consensus on definition and diagnosis: report of the European Working Group on Sarcopenia in Older People. Age Ageing 2010;39:412–423. 20392703 10.1093/ageing/afq034 PMC2886201

[14] Evans WJ . What is sarcopenia? The Journals of Gerontology Series A, Biological Sciences and Medical Sciences 1995;50:5–8. 10.1093/gerona/50a.special_issue.5 7493218

[15] Lin J , Lopez EF , Jin Y , Van Remmen H , Bauch T , Han HC , et al. Age‐related cardiac muscle sarcopenia: combining experimental and mathematical modeling to identify mechanisms. Exp Gerontol 2008;43:296–306. 18221848 10.1016/j.exger.2007.12.005 PMC2323436

[16] Thompson LV . Age‐related muscle dysfunction. Exp Gerontol 2009;44:106–111. 18657920 10.1016/j.exger.2008.05.003 PMC4074082

[17] Fielding RA , Vellas B , Evans WJ , Bhasin S , Morley JE , Newman AB , et al. Sarcopenia: an undiagnosed condition in older adults. Current consensus definition: prevalence, etiology, and consequences. International working group on sarcopenia. J Am Med Dir Assoc 2011;12:249–256. 21527165 10.1016/j.jamda.2011.01.003 PMC3377163

[18] Morley JE . Sarcopenia: diagnosis and treatment. J Nutr Health Aging 2008;12:452–456.1861522610.1007/BF02982705

[19] Ali S , Garcia JM . Sarcopenia, cachexia and aging: diagnosis, mechanisms and therapeutic options—a mini‐review. Gerontology 2014;60:294–305. 24731978 10.1159/000356760 PMC4112511

[20] Hallgren M , Herring MP , Owen N , Dunstan D , Ekblom Ö , Helgadottir B , et al. Exercise, physical activity, and sedentary behavior in the treatment of depression: broadening the scientific perspectives and clinical opportunities. Front Psych 2016;7:36. 10.3389/fpsyt.2016.00036 PMC4786540 27014101

[21] Kim JK , Choi SR , Choi MJ , Kim SG , Lee YK , Noh JW , et al. Prevalence of and factors associated with sarcopenia in elderly patients with end‐stage renal disease. Clin Nutr (Edinburgh, Scotland) 2014;33:64–68. 10.1016/j.clnu.2013.04.002 23631844

[22] Patino‐Hernandez D , David‐Pardo DG , Borda MG , Pérez‐Zepeda MU , Cano‐Gutiérrez C . Association of fatigue with sarcopenia and its elements: a secondary analysis of SABE‐Bogotá. Gerontol Geriatr Med 2017;3:2333721417703734. 28474000 10.1177/2333721417703734 PMC5407660

[23] Chang KV , Hsu TH , Wu WT , Huang KC , Han DS . Is sarcopenia associated with depression? A systematic review and meta‐analysis of observational studies. Age Ageing 2017;46:738–746. 28633395 10.1093/ageing/afx094

[24] Stang A . Critical evaluation of the Newcastle–Ottawa Scale for the assessment of the quality of nonrandomized studies in meta‐analyses. Eur J Epidemiol 2010;25:603–605. 20652370 10.1007/s10654-010-9491-z

[25] Tong X , Wang D , Liu S , Ma Y , Li Z , Tian P , et al. The YKL‐40 protein is a potential biomarker for COPD: a meta‐analysis and systematic review. Int J Chron Obstruct Pulmon Dis 2018;13:409–418. 29430175 10.2147/COPD.S152655 PMC5796800

[26] Brockwell SE , Gordon IR . A comparison of statistical methods for meta‐analysis. Stat Med 2001;20:825–840. 11252006 10.1002/sim.650

[27] World Health Organisation . Obesity and overweight. https://www.who.int/news‐room/fact‐sheets/detail/obesity‐and‐overweight (accessed 11 April 2021).

[28] Endo T , Akai K , Kijima T , Kitahara S , Abe T , Takeda M , et al. An association analysis between hypertension, dementia, and depression and the phases of pre‐sarcopenia to sarcopenia: a cross‐sectional analysis. PLoS ONE 2021;16:e0252784. 34292967 10.1371/journal.pone.0252784 PMC8297796

[29] Kitamura A , Seino S , Abe T , Nofuji Y , Yokoyama Y , Amano H , et al. Sarcopenia: prevalence, associated factors, and the risk of mortality and disability in Japanese older adults. J Cachexia Sarcopenia Muscle 2021;12:30–38. 33241660 10.1002/jcsm.12651 PMC7890144

[30] Yuenyongchaiwat K , Jongritthiporn S , Somsamarn K , Sukkho O , Pairojkittrakul S , Traitanon O . Depression and low physical activity are related to sarcopenia in hemodialysis: a single‐center study. PeerJ 2021;9:e11695. 34249515 10.7717/peerj.11695 PMC8253107

[31] Fábrega‐Cuadros R , Cruz‐Díaz D , Martínez‐Amat A , Aibar‐Almazán A , Redecillas‐Peiró MT , Hita‐Contreras F . Associations of sleep and depression with obesity and sarcopenia in middle‐aged and older adults. Maturitas 2020;142:1–7. 33158481 10.1016/j.maturitas.2020.06.019

[32] Yuenyongchaiwat K , Boonsinsukh R . Sarcopenia and its relationships with depression, cognition, and physical activity in Thai community‐dwelling older adults. Curr Gerontol Geriatr Res 2020;2020:8041489. 33424964 10.1155/2020/8041489 PMC7773447

[33] Olgun Yazar H , Yazar T . Prevalence of sarcopenia in patients with geriatric depression diagnosis. Ir J Med Sci 2019;188:931–938. 30610679 10.1007/s11845-018-01957-7

[34] Kilavuz A , Meseri R , Savas S , Simsek H , Sahin S , Bicakli DH , et al. Association of sarcopenia with depressive symptoms and functional status among ambulatory community‐dwelling elderly. Arch Gerontol Geriatr 2018;76:196–201. 29550658 10.1016/j.archger.2018.03.003

[35] Szlejf C , Suemoto CK , Brunoni AR , Viana MC , Moreno AB , Matos SMA , et al. Depression is associated with sarcopenia due to low muscle strength: results from the ELSA‐Brasil Study. J Am Med Dir Assoc 2019;20:1641–1646. 30409492 10.1016/j.jamda.2018.09.020

[36] Hayashi T , Umegaki H , Makino T , Cheng XW , Shimada H , Kuzuya M . Association between sarcopenia and depressive mood in urban‐dwelling older adults: a cross‐sectional study. Geriatr Gerontol Int 2019;19:508–512. 30884107 10.1111/ggi.13650

[37] Wang H , Hai S , Liu Y , Cao L , Liu Y , Liu P , et al. Association between depressive symptoms and sarcopenia in older Chinese community‐dwelling individuals. Clin Interv Aging 2018;13:1605–1611. 30233157 10.2147/CIA.S173146 PMC6130547

[38] Lee I , Cho J , Hong H , Jin Y , Kim D , Kang H . Sarcopenia is associated with cognitive impairment and depression in elderly Korean Women. Iran J Public Health 2018;47:327–334. 29845019 PMC5971168

[39] Sugimoto T , Ono R , Murata S , Saji N , Matsui Y , Niida S , et al. Prevalence and associated factors of sarcopenia in elderly subjects with amnestic mild cognitive impairment or Alzheimer disease. Curr Alzheimer Res 2016;13:718–726. 26863996 10.2174/1567205013666160211124828

[40] Ishii S , Chang C , Tanaka T , Kuroda A , Tsuji T , Akishita M , et al. The association between sarcopenic obesity and depressive symptoms in older Japanese adults. PLoS ONE 2016;11:e0162898. 27627756 10.1371/journal.pone.0162898 PMC5023182

[41] Huang CY , Hwang AC , Liu LK , Lee WJ , Chen LY , Peng LN , et al. Association of dynapenia, sarcopenia, and cognitive impairment among community‐dwelling older Taiwanese. Rejuvenation Res 2016;19:71–78. 26165544 10.1089/rej.2015.1710

[42] Alexandre Tda S , Duarte YA , Santos JL , Wong R , Lebrão ML . Prevalence and associated factors of sarcopenia among elderly in Brazil: findings from the SABE study. J Nutr Health Aging 2014;18:284–290.2462675610.1007/s12603-013-0413-0

[43] Hsu YH , Liang CK , Chou MY , Liao MC , Lin YT , Chen LK , et al. Association of cognitive impairment, depressive symptoms and sarcopenia among healthy older men in the veterans retirement community in southern Taiwan: a cross‐sectional study. Geriatr Gerontol Int 2014;14:102–108. 24450567 10.1111/ggi.12221

[44] Landi F , Liperoti R , Russo A , Giovannini S , Tosato M , Capoluongo E , et al. Sarcopenia as a risk factor for falls in elderly individuals: results from the ilSIRENTE study. Clin Nutr (Edinburgh, Scotland) 2012;31:652–658. 10.1016/j.clnu.2012.02.007 22414775

[45] Chen LK , Woo J , Assantachai P , Auyeung TW , Chou MY , Iijima K , et al. Asian Working Group for Sarcopenia: 2019 Consensus Update on Sarcopenia Diagnosis and Treatment. J Am Med Dir Assoc 2020;21:300–7.e2. 32033882 10.1016/j.jamda.2019.12.012

[46] Chen LK , Liu LK , Woo J , Assantachai P , Auyeung TW , Bahyah KS , et al. Sarcopenia in Asia: consensus report of the Asian Working Group for Sarcopenia. J Am Med Dir Assoc 2014;15:95–101. 24461239 10.1016/j.jamda.2013.11.025

[47] Tanimoto Y , Watanabe M , Sun W , Hirota C , Sugiura Y , Kono R , et al. Association between muscle mass and disability in performing instrumental activities of daily living (IADL) in community‐dwelling elderly in Japan. Arch Gerontol Geriatr 2012;54:e230–e233. 21831461 10.1016/j.archger.2011.06.015

[48] McLean RR , Kiel DP . Developing consensus criteria for sarcopenia: an update. J Bone Miner Res 2015;30:588–592. 25735999 10.1002/jbmr.2492

[49] Chien MY , Huang TY , Wu YT . Prevalence of sarcopenia estimated using a bioelectrical impedance analysis prediction equation in community‐dwelling elderly people in Taiwan. J Am Geriatr Soc 2008;56:1710–1715. 18691288 10.1111/j.1532-5415.2008.01854.x

[50] Newman AB , Kupelian V , Visser M , Simonsick E , Goodpaster B , Nevitt M , et al. Sarcopenia: alternative definitions and associations with lower extremity function. J Am Geriatr Soc 2003;51:1602–1609. 14687390 10.1046/j.1532-5415.2003.51534.x

[51] Delmonico MJ , Harris TB , Lee JS , Visser M , Nevitt M , Kritchevsky SB , et al. Alternative definitions of sarcopenia, lower extremity performance, and functional impairment with aging in older men and women. J Am Geriatr Soc 2007;55:769–774. 17493199 10.1111/j.1532-5415.2007.01140.x

[52] Antonelli Incalzi R , Landi F , Cipriani L , Bruno E , Pagano F , Gemma A , et al. Nutritional assessment: a primary component of multidimensional geriatric assessment in the acute care setting. J Am Geriatr Soc 1996;44:166–174. 8576507 10.1111/j.1532-5415.1996.tb02434.x

[53] Lauretani F , Russo CR , Bandinelli S , Bartali B , Cavazzini C , Di Iorio A , et al. Age‐associated changes in skeletal muscles and their effect on mobility: an operational diagnosis of sarcopenia. J Appl Physiol (Bethesda, Md: 1985) 2003;95:1851–1860. 10.1152/japplphysiol.00246.2003 14555665

[54] McLean RR , Shardell MD , Alley DE , Cawthon PM , Fragala MS , Harris TB , et al. Criteria for clinically relevant weakness and low lean mass and their longitudinal association with incident mobility impairment and mortality: the foundation for the National Institutes of Health (FNIH) sarcopenia project. J Gerontol A Biol Sci Med Sci 2014;69:576–583. 24737560 10.1093/gerona/glu012 PMC3991140

[55] Liu LK , Lee WJ , Liu CL , Chen LY , Lin MH , Peng LN , et al. Age‐related skeletal muscle mass loss and physical performance in Taiwan: implications to diagnostic strategy of sarcopenia in Asia. Geriatr Gerontol Int 2013;13:964–971. 23452090 10.1111/ggi.12040

[56] Wu SW , Wu SF , Liang HW , Wu ZT , Huang S . Measuring factors affecting grip strength in a Taiwan Chinese population and a comparison with consolidated norms. Appl Ergon 2009;40:811–815. 18947819 10.1016/j.apergo.2008.08.006

[57] Dodds RM , Syddall HE , Cooper R , Benzeval M , Deary IJ , Dennison EM , et al. Grip strength across the life course: normative data from twelve British studies. PLoS ONE 2014;9:e113637. 25474696 10.1371/journal.pone.0113637 PMC4256164

[58] Andreoli A , Scalzo G , Masala S , Tarantino U , Guglielmi G . Body composition assessment by dual‐energy X‐ray absorptiometry (DXA). Radiol Med 2009;114:286–300. 19266259 10.1007/s11547-009-0369-7

[59] Meeuwsen S , Horgan GW , Elia M . The relationship between BMI and percent body fat, measured by bioelectrical impedance, in a large adult sample is curvilinear and influenced by age and sex. Clin Nutr (Edinburgh, Scotland) 2010;29:560–566. 10.1016/j.clnu.2009.12.011 20359792

[60] Landi F , Liperoti R , Fusco D , Mastropaolo S , Quattrociocchi D , Proia A , et al. Prevalence and risk factors of sarcopenia among nursing home older residents. J Gerontol A Biol Sci Med Sci 2012;67:48–55. 21393423 10.1093/gerona/glr035

[61] Achamrah N , Colange G , Delay J , Rimbert A , Folope V , Petit A , et al. Comparison of body composition assessment by DXA and BIA according to the body mass index: a retrospective study on 3655 measures. PLoS ONE 2018;13:e0200465. 30001381 10.1371/journal.pone.0200465 PMC6042744

[62] Krishnamoorthy Y , Rajaa S , Rehman T . Diagnostic accuracy of various forms of Geriatric Depression Scale for screening of depression among older adults: systematic review and meta‐analysis. Arch Gerontol Geriatr 2020;87:104002. 31881393 10.1016/j.archger.2019.104002

[63] Pocklington C , Gilbody S , Manea L , McMillan D . The diagnostic accuracy of brief versions of the Geriatric Depression Scale: a systematic review and meta‐analysis. Int J Geriatr Psychiatry 2016;31:837–857. 26890937 10.1002/gps.4407

[64] Koltun DO , Marquart TA , Shenk KD , Elzein E , Li Y , Nguyen M , et al. New fatty acid oxidation inhibitors with increased potency lacking adverse metabolic and electrophysiological properties. Bioorg Med Chem Lett 2004;14:549–552. 14698201 10.1016/j.bmcl.2003.09.093

[65] Binder DK , Scharfman HE . Brain‐derived neurotrophic factor. Growth Factors (Chur, Switzerland) 2004;22:123–131. 10.1080/08977190410001723308 PMC2504526 15518235

[66] Campbell S , Marriott M , Nahmias C , MacQueen GM . Lower hippocampal volume in patients suffering from depression: a meta‐analysis. Am J Psychiatry 2004;161:598–607. 15056502 10.1176/appi.ajp.161.4.598

[67] Brach JS , FitzGerald S , Newman AB , Kelsey S , Kuller L , VanSwearingen JM , et al. Physical activity and functional status in community‐dwelling older women: a 14‐year prospective study. Arch Intern Med 2003;163:2565–2571. 14638556 10.1001/archinte.163.21.2565

[68] Chen X , Guo J , Han P , Fu L , Jia L , Yu H , et al. Twelve‐month incidence of depressive symptoms in suburb‐dwelling Chinese older adults: role of sarcopenia. J Am Med Dir Assoc 2019;20:64–69. 30268631 10.1016/j.jamda.2018.07.017

[69] Luppino FS , de Wit LM , Bouvy PF , Stijnen T , Cuijpers P , Penninx BW , et al. Overweight, obesity, and depression: a systematic review and meta‐analysis of longitudinal studies. Arch Gen Psychiatry 2010;67:220–229. 20194822 10.1001/archgenpsychiatry.2010.2

[70] Weber‐Hamann B , Hentschel F , Kniest A , Deuschle M , Colla M , Lederbogen F , et al. Hypercortisolemic depression is associated with increased intra‐abdominal fat. Psychosom Med 2002;64:274–277. 11914443 10.1097/00006842-200203000-00010

[71] Kim NH , Kim HS , Eun CR , Seo JA , Cho HJ , Kim SG , et al. Depression is associated with sarcopenia, not central obesity, in elderly Korean men. J Am Geriatr Soc 2011;59:2062–2068. 22092258 10.1111/j.1532-5415.2011.03664.x

[72] Crisp AH , McGuiness B . Jolly fat: relation between obesity and psychoneurosis in general population. Br Med J 1976;1:7–9. 1247732 10.1136/bmj.1.6000.7 PMC1638245

[73] Palinkas LA , Wingard DL , Barrett‐Connor E . Depressive symptoms in overweight and obese older adults: a test of the “jolly fat” hypothesis. J Psychosom Res 1996;40:59–66. 8730645 10.1016/0022-3999(95)00542-0

[74] Kuriyama S , Koizumi Y , Matsuda‐Ohmori K , Seki T , Shimazu T , Hozawa A , et al. Obesity and depressive symptoms in elderly Japanese: the Tsurugaya Project. J Psychosom Res 2006;60:229–235. 16516653 10.1016/j.jpsychores.2005.07.010

[75] Roberts RE , Strawbridge WJ , Deleger S , Kaplan GA . Are the fat more jolly? Ann Behav Med 2002;24:169–180. 12173674 10.1207/S15324796ABM2403_02

[76] Zhao G , Ford ES , Dhingra S , Li C , Strine TW , Mokdad AH . Depression and anxiety among US adults: associations with body mass index. Int J Obes (2005) 2009;33:257–266. 10.1038/ijo.2008.268 19125163

[77] Lee JS , Auyeung TW , Kwok T , Lau EM , Leung PC , Woo J . Associated factors and health impact of sarcopenia in older chinese men and women: a cross‐sectional study. Gerontology 2007;53:404–410. 17700027 10.1159/000107355

[78] Byeon CH , Kang KY , Kang SH , Kim HK , Bae EJ . Sarcopenia is not associated with depression in Korean adults: results from the 2010–2011 Korean National Health and Nutrition Examination Survey. Korean J Fam Med 2016;37:37–43. 26885321 10.4082/kjfm.2016.37.1.37 PMC4754285

[79] Venant V , Pouget M , Lahaye C , Gentes E , Pereira B , Lambert C , et al. Depression severity as a risk factor of sarcopenic obesity in morbidly obese patients. J Nutr Health Aging 2019;23:761–767.3156003610.1007/s12603-019-1218-6

[80] Gao L , Jiang J , Yang M , Hao Q , Luo L , Dong B . Prevalence of sarcopenia and associated factors in chinese community‐dwelling elderly: comparison between rural and urban areas. J Am Med Dir Assoc 2015;16:1003.e1–1003.e6. 10.1016/j.jamda.2015.07.020 26385304

[81] Huo YR , Suriyaarachchi P , Gomez F , Curcio CL , Boersma D , Muir SW , et al. Phenotype of osteosarcopenia in older individuals with a history of falling. J Am Med Dir Assoc 2015;16:290–295. 25512216 10.1016/j.jamda.2014.10.018

[82] Cho Y , Shin SY , Shin MJ . Sarcopenic obesity is associated with lower indicators of psychological health and quality of life in Koreans. Nutr Res (New York, NY) 2015;35:384–392. 10.1016/j.nutres.2015.04.002 25931418

[83] Vasconcelos KS , Dias JM , Bastone Ade C , Vieira RA , Andrade AC , Perracini MR , et al. Handgrip strength cutoff points to identify mobility limitation in community‐dwelling older people and associated factors. J Nutr Health Aging 2016;20:306–315.2689258010.1007/s12603-015-0584-y

[84] Kim YH , Kim KI , Paik NJ , Kim KW , Jang HC , Lim JY . Muscle strength: a better index of low physical performance than muscle mass in older adults. Geriatr Gerontol Int 2016;16:577–585. 26017097 10.1111/ggi.12514

[85] Hamer M , Batty GD , Kivimaki M . Sarcopenic obesity and risk of new onset depressive symptoms in older adults: English Longitudinal Study of Ageing. Int J Obes (2005) 2015;39:1717–1720. 10.1038/ijo.2015.124 PMC4722238 26122029

[86] Fukumori N , Yamamoto Y , Takegami M , Yamazaki S , Onishi Y , Sekiguchi M , et al. Association between hand‐grip strength and depressive symptoms: locomotive syndrome and health outcomes in Aizu Cohort Study (LOHAS). Age Ageing 2015;44:592–598. 25712514 10.1093/ageing/afv013

[87] Alkan Melikoğlu M . Presarcopenia and its impact on disability in female patients with rheumatoid arthritis. Arch Rheumatol 2017;32:53–59. 30375535 10.5606/ArchRheumatol.2017.6078 PMC6190944

