# Supplementary material for: Prevalence of depression in patients with sarcopenia and correlation between the two diseases: systematic review and meta‐analysis
Source: J Cachexia Sarcopenia Muscle. 2022 Jan 8;13(1):128–44. doi: 10.1002/jcsm.12908 (PMC8818614; doi:10.1002/jcsm.12908)
Supplement: Supplementary file 1 — Table S1. The reasons for the exclusion of full‐text articles. [file JCSM-13-128-s001.docx]

Table S1: The reasons for the exclusion of full-text articles

| Study | Reason for the exclusion |
| --- | --- |
| Kirk (2021)[1] | Not use acceptable diagnostic criteria: sarcopenia diagnosed by grip strength and gait speed |
| Nipp (2018)[2] | Not use acceptable diagnostic criteria: sarcopenia diagnosed by CT scans |
| Byeon (2016)[3] | Not use acceptable diagnostic criteria: sarcopenia diagnosed by low muscle mass. |
| Ida (2017)[4] | Not use acceptable diagnostic criteria: sarcopenia diagnosed by SARC-F-J |
| Kurita (2021)[5] | Not use acceptable diagnostic criteria: sarcopenia diagnosed by SARC-F-J |
| Souza（2021）[6] | Not use acceptable diagnostic criteria: sarcopenia diagnosed by Five times sit-to-stand test |
| Fujisawa (2021)[7] | Not reported specific prevalence data and ORs between sarcopenia and depression |
| Delibaş (2021)[8] | Not reported specific prevalence data and ORs between sarcopenia and depression |
| [Kim](https://pubmed.ncbi.nlm.nih.gov/?term=Kim+JH&cauthor_id=27051246) (2016)[9] | Not reported specific prevalence data and ORs between sarcopenia and depression |
| [Nishikawa](https://pubmed.ncbi.nlm.nih.gov/?term=Nishikawa+H&cauthor_id=31072067) (2019)[10] | Not reported specific prevalence data and ORs between sarcopenia and depression |
| [Wang](https://pubmed.ncbi.nlm.nih.gov/?term=Wang+LT&cauthor_id=34179935) (2021)[11] | Not reported specific prevalence data and ORs between sarcopenia and depression |
| [Chen](https://pubmed.ncbi.nlm.nih.gov/?term=Chen+X&cauthor_id=33714172) (2021)[12] | Not reported specific prevalence data and ORs between sarcopenia and depression |
| [Chen](https://pubmed.ncbi.nlm.nih.gov/?term=Chen+X&cauthor_id=30268631) (2019)[13] | Not reported specific prevalence data and ORs between sarcopenia and depression |
| [Hamer](https://pubmed.ncbi.nlm.nih.gov/?term=Hamer+M&cauthor_id=26122029)(2015)[14] | Not use acceptable diagnostic criteria: sarcopenia diagnosed by grip strength |
| Liu (2020)[15] | Not reported specific prevalence data and ORs between sarcopenia and depression |
| Kim (2011)[16] | Not reported specific prevalence data and ORs between sarcopenia and depression |
| Chen (2020)[17] | Not reported specific prevalence data and ORs between sarcopenia and depression () |
| Samper-Ternent (2017)[18] | Not reported specific prevalence data and ORs between sarcopenia and depression) |
| [Wu](https://pubmed.ncbi.nlm.nih.gov/?term=Wu+H&cauthor_id=27272735) (2017)[19] | Not reported specific prevalence data and ORs between sarcopenia and depression |
| [Kahl](https://pubmed.ncbi.nlm.nih.gov/?term=Kahl+KG&cauthor_id=28132777) (2017)[20] | Not reported specific prevalence data and ORs between sarcopenia and depression |
| [İlhan](https://pubmed.ncbi.nlm.nih.gov/?term=%C4%B0lhan+B&cauthor_id=30697631) (2019)[21] | Not reported specific prevalence data and ORs between sarcopenia and depression |
| [Gariballa](https://pubmed.ncbi.nlm.nih.gov/?term=Gariballa+S&cauthor_id=28527135) (2018)[22] | Not reported specific prevalence data and ORs between sarcopenia and depression |

References:

1. Kirk B, Zanker J, Bani Hassan E, Bird S, Brennan-Olsen S, Duque G. Sarcopenia Definitions and Outcomes Consortium (SDOC) Criteria are Strongly Associated With Malnutrition, Depression, Falls, and Fractures in High-Risk Older Persons. Journal of the American Medical Directors Association. 2021;22:741-5.

2. Nipp RD, Fuchs G, El-Jawahri A, Mario J, Troschel FM, Greer JA, et al. Sarcopenia Is Associated with Quality of Life and Depression in Patients with Advanced Cancer. The oncologist. 2018;23:97-104.

3. Byeon CH, Kang KY, Kang SH, Kim HK, Bae EJ. Sarcopenia Is Not Associated with Depression in Korean Adults: Results from the 2010-2011 Korean National Health and Nutrition Examination Survey. Korean journal of family medicine. 2016;37:37-43.

4. Ida S, Murata K, Nakai M, Ito S, Malmstrom TK, Ishihara Y, et al. Relationship between sarcopenia and depression in older patients with diabetes: An investigation using the Japanese version of SARC-F. Geriatrics & gerontology international. 2018;18:1318-22.

5. Kurita N, Wakita T, Fujimoto S, Yanagi M, Koitabashi K, Suzuki T, et al. Hopelessness and Depression Predict Sarcopenia in Advanced CKD and Dialysis: A Multicenter Cohort Study. The journal of nutrition, health & aging. 2021;25:593-9.

6. de Souza LF, Fontanela LC, Gonçalves C, Mendrano AL, Freitas MA, Danielewicz AL, et al. Cognitive and behavioral factors associated to probable sarcopenia in community-dwelling older adults. Experimental aging research. 2021:1-14.

7. Fujisawa C, Umegaki H, Sugimoto T, Samizo S, Huang CH, Fujisawa H, et al. Mild hyponatremia is associated with low skeletal muscle mass, physical function impairment, and depressive mood in the elderly. BMC geriatrics. 2021;21:15.

8. Delibaş DH, Eşkut N, İlhan B, Erdoğan E, Top Kartı D, Yılmaz Küsbeci Ö, et al. Clarifying the relationship between sarcopenia and depression in geriatric outpatients. The aging male : the official journal of the International Society for the Study of the Aging Male. 2021;24:29-36.

9. Nishikawa H, Enomoto H, Yoh K, Iwata Y, Sakai Y, Kishino K, et al. Association between Sarcopenia and Depression in Patients with Chronic Liver Diseases. Journal of clinical medicine. 2019;8.

10. Kim JH, Kim DH, Park YS. Body Composition, Sarcopenia, and Suicidal Ideation in Elderly Koreans: Hallym Aging Study. Journal of Korean medical science. 2016;31:604-10.

11. Wang LT, Huang WC, Hung YC, Park JH. Association between Depressive Symptoms and Risk of Sarcopenia in Taiwanese Older Adults. The journal of nutrition, health & aging. 2021;25:790-4.

12. Chen X, Han P, Yu X, Zhang Y, Song P, Liu Y, et al. Relationships between sarcopenia, depressive symptoms, and mild cognitive impairment in Chinese community-dwelling older adults. Journal of affective disorders. 2021;286:71-7.

13. Chen X, Guo J, Han P, Fu L, Jia L, Yu H, et al. Twelve-Month Incidence of Depressive Symptoms in Suburb-Dwelling Chinese Older Adults: Role of Sarcopenia. Journal of the American Medical Directors Association. 2019;20:64-9.

14. Hamer M, Batty GD, Kivimaki M. Sarcopenic obesity and risk of new onset depressive symptoms in older adults: English Longitudinal Study of Ageing. International journal of obesity (2005). 2015;39:1717-20.

15. Liu X, Hao Q, Yue J, Hou L, Xia X, Zhao W, et al. Sarcopenia, Obesity and Sarcopenia Obesity in Comparison: Prevalence, Metabolic Profile, and Key Differences: Results from WCHAT Study. The journal of nutrition, health & aging. 2020;24:429-37.

16. Kim NH, Kim HS, Eun CR, Seo JA, Cho HJ, Kim SG, et al. Depression is associated with sarcopenia, not central obesity, in elderly korean men. Journal of the American Geriatrics Society. 2011;59:2062-8.

17. Chen L, Sheng Y, Qi H, Tang T, Yu J, Lv S. Correlation of sarcopenia and depressive mood in older community dwellers: a cross-sectional observational study in China. BMJ open. 2020;10:e038089.

18. Samper-Ternent R, Reyes-Ortiz C, Ottenbacher KJ, Cano CA. Frailty and sarcopenia in Bogotá: results from the SABE Bogotá Study. Aging clinical and experimental research. 2017;29:265-72.

19. Wu H, Yu B, Meng G, Liu F, Guo Q, Wang J, et al. Both muscle mass and muscle strength are inversely associated with depressive symptoms in an elderly Chinese population. International journal of geriatric psychiatry. 2017;32:769-78.

20. Kahl KG, Utanir F, Schweiger U, Krüger TH, Frieling H, Bleich S, et al. Reduced muscle mass in middle-aged depressed patients is associated with male gender and chronicity. Progress in neuro-psychopharmacology & biological psychiatry. 2017;76:58-64.

21. İlhan B, Bahat G, Erdoğan T, Kılıç C, Karan MA. Anorexia Is Independently Associated with Decreased Muscle Mass and Strength in Community Dwelling Older Adults. The journal of nutrition, health & aging. 2019;23:202-6.

22. Gariballa S, Alessa A. Association between muscle function, cognitive state, depression symptoms and quality of life of older people: evidence from clinical practice. Aging clinical and experimental research. 2018;30:351-7.
