# Supplementary material for: Prevalence of depression in patients with sarcopenia and correlation between the two diseases: systematic review and meta‐analysis
Source: J Cachexia Sarcopenia Muscle. 2022 Jan 8;13(1):128–44. doi: 10.1002/jcsm.12908 (PMC8818614; doi:10.1002/jcsm.12908)
Supplement: Supplementary file 2 — Table S2. Risk of bias of the included studies using the Newcastle–Ottawa Scale. [file JCSM-13-128-s004.docx]

Table S2: Risk of bias of the included studies using the Newcastle-Ottawa Scale.

| First author and year | Selection | | | | Comparability | Outcome | | |
| --- | --- | --- | --- | --- | --- | --- | --- | --- |
|  | Q1 | Q2 | Q3 | Q4 | Q1 | Q1 | Q2 | Q3 |
| Endo (2021) | * | * | * | * | ** | ** | * |  |
| Kitamura (2021) | * | * | * | * | ** | ** | * |  |
| Olgun Yazar (2019) | * | * | * | * | * | ** | * |  |
| Kilavuz (2018) | * | * | * | * | ** | ** | * |  |
| Szlejf (2018) | * | * | * | * | ** | ** | * |  |
| Hayashi (2018) | * | * | * | * | ** | ** | * |  |
| Wang (2018) | * | * | * | * | ** | ** | * |  |
| Sugimoto (2016) | * | * |  | * | ** | ** | * |  |
| Ishii (2016) | * | * | * | * | ** | ** | * |  |
| Huang (2015) | * | * | * | * | * | ** | * |  |
| Alexandre (2014) | * | * | * | * | * | ** | * |  |
| Ying-Hsin (2014) | * | * | * | * | ** | ** | * |  |
| Landi (2012) | * | * | * | * | * |  |  |  |
| Yuenyongchaiwat (2021) | * | * |  | * | ** | ** | * |  |
| Fábrega-Cuadros (2020) | * | * | * | * | ** | ** | * |  |
| Yuenyongchaiwat (2020) | * | * | * | * | ** | ** | * |  |
| Lee (2018) | * | * | * | * | ** | ** | * |  |
| Patino-Hernandez (2017) | * | * | * | * | ** | ** | * |  |
| Kim (2013) | * | * |  | * | ** | ** | * |  |

NEWCASTLE - OTTAWA QUALITY ASSESSMENT SCALE

CASE CONTROL STUDIES

Note: A study can be awarded a maximum of one star for each numbered item within the Selection and Exposure categories. A maximum of two stars can be given for Comparability.

Selection

1) Is the case definition adequate?

a) yes, with independent validation 

b) yes, eg record linkage or based on self reports

c) no description

2) Representativeness of the cases

a) consecutive or obviously representative series of cases 

b) potential for selection biases or not stated

3) Selection of Controls

a) community controls 

b) hospital controls

c) no description

4) Definition of Controls

a) no history of disease (endpoint) 

b) no description of source

Comparability

1) Comparability of cases and controls on the basis of the design or analysis

a) study controls for ______________ (Select the most important factor.) 

b) study controls for any additional factor. (This criteria could be modified to indicate specific control for a second important factor.)

Exposure

1) Ascertainment of exposure

a) secure record (eg surgical records) 

b) structured interview where blind to case/control status 

c) interview not blinded to case/control status

d) written self report or medical record only

e) no description

2) Same method of ascertainment for cases and controls

a) yes 

b) no

3) Non-Response rate

a) same rate for both groups 

b) non respondents described

c) rate different and no designation
