# Supplementary figures and images for: Prevalence of depression in patients with sarcopenia and correlation between the two diseases: systematic review and meta‐analysis
Source: J Cachexia Sarcopenia Muscle. 2022 Jan 8;13(1):128–44. doi: 10.1002/jcsm.12908 (PMC8818614; doi:10.1002/jcsm.12908)

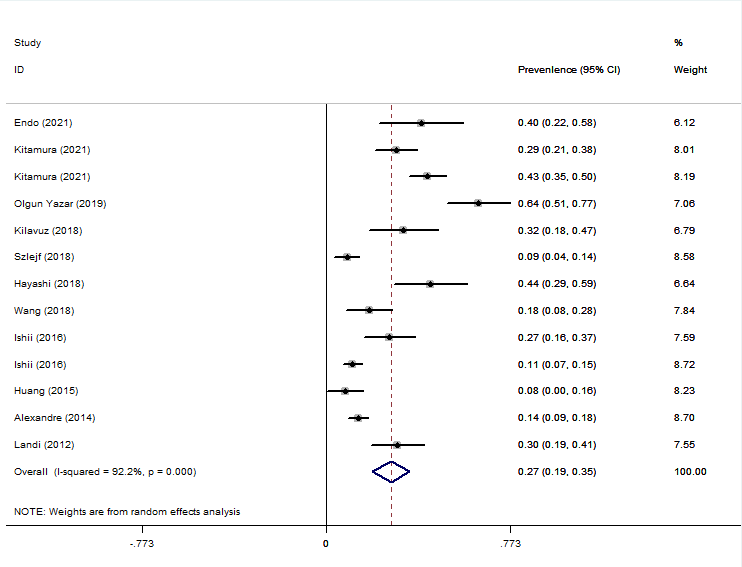

Supplement: Supplementary file 3 — Figure S1. The sensitivity analysis for prevalence of depression in sarcopenia. [file JCSM-13-128-s003.tif]

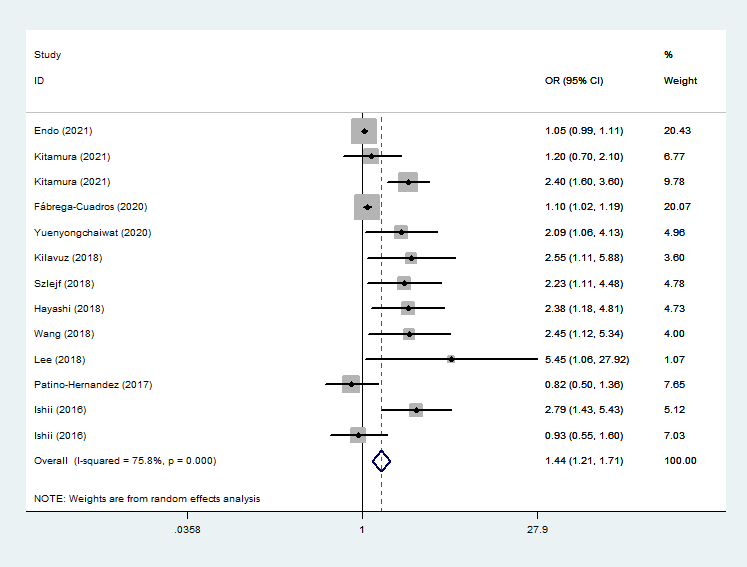

Supplement: Supplementary file 4 — Figure S2. The sensitivity analysis for adjusted ORs between sarcopenia and depression. [file JCSM-13-128-s002.tif]
